# A Multiscale Vibrational Spectroscopic Approach for Identification and Biochemical Characterization of Pollen

**DOI:** 10.1371/journal.pone.0137899

**Published:** 2015-09-16

**Authors:** Murat Bağcıoğlu, Boris Zimmermann, Achim Kohler

**Affiliations:** 1 Department of Mathematical Sciences and Technology, Faculty of Environmental Science and Technology, Norwegian University of Life Sciences, Ås, Norway; 2 Nofima AS, Ås, Norway; Glasgow University, UNITED KINGDOM

## Abstract

**Background:**

Analysis of pollen grains reveals valuable information on biology, ecology, forensics, climate change, insect migration, food sources and aeroallergens. Vibrational (infrared and Raman) spectroscopies offer chemical characterization of pollen via identifiable spectral features without any sample pretreatment. We have compared the level of chemical information that can be obtained by different multiscale vibrational spectroscopic techniques.

**Methodology:**

Pollen from 15 different species of Pinales (conifers) were measured by seven infrared and Raman methodologies. In order to obtain infrared spectra, both reflectance and transmission measurements were performed on ground and intact pollen grains (bulk measurements), in addition, infrared spectra were obtained by microspectroscopy of multigrain and single pollen grain measurements. For Raman microspectroscopy measurements, spectra were obtained from the same pollen grains by focusing two different substructures of pollen grain. The spectral data from the seven methodologies were integrated into one data model by the Consensus Principal Component Analysis, in order to obtain the relations between the molecular signatures traced by different techniques.

**Results:**

The vibrational spectroscopy enabled biochemical characterization of pollen and detection of phylogenetic variation. The spectral differences were clearly connected to specific chemical constituents, such as lipids, carbohydrates, carotenoids and sporopollenins. The extensive differences between pollen of *Cedrus* and the rest of Pinaceae family were unambiguously connected with molecular composition of sporopollenins in pollen grain wall, while pollen of *Picea* has apparently higher concentration of carotenoids than the rest of the family. It is shown that vibrational methodologies have great potential for systematic collection of data on ecosystems and that the obtained phylogenetic variation can be well explained by the biochemical composition of pollen. Out of the seven tested methodologies, the best taxonomical differentiation of pollen was obtained by infrared measurements on bulk samples, as well as by Raman microspectroscopy measurements of the corpus region of the pollen grain. Raman microspectroscopy measurements indicate that measurement area, as well as the depth of focus, can have crucial influence on the obtained data.

## Introduction

Pollen grains are characteristic to plant families, genera or even species, thus revealing valuable information on ecology, forensics, climate change, insect migration, food sources and aeroallergens. They are extremely resilient to decay, and pollen microfossils can have well-preserved morphology for millions of years. Thus in palaeoecology, palaeobotany, biostratigraphy and biogeography, identification of pollen microfossils is important for the reconstruction of past flora, population sizes and terrestrial communities. Moreover, microfossil data is vital for the reconstruction of past environments and for the understanding of the causes of environmental changes.

The main drawback of pollen analysis is that pollen grains are still studied by measuring their morphology employing conventional microscopy. Contemporary pollen identification is predominantly performed by optical microscopy. Thus, routine analysis is labour-intensive and uneconomical, where thousands of discrete pollen grains are examined by an experienced scientist after complex chemical pre-treatment of samples. In addition to optical microscopy, different microscopic techniques have been attempted for measurement of pollen, such as differential interference contrast microscopy, phase contrast microscopy, fluorescence microscopy, scanning electron microscopy, confocal laser scanning microscopy and other alternative methods for obtaining detailed morphology of pollen [1]. Unfortunately, these methods are time-consuming and expensive and provide only identification based on morphological analysis, while chemical characterization of samples cannot be obtained. Alternative analyses, such as biochemical and sequencing methods, are even more complex and expensive [2].

Vibrational (infrared and Raman) spectroscopies offer an alternative approach to pollen analysis. Instead of morphological characterization of grains, in vibrational spectroscopy pollen analysis is based on chemical characterization via identifiable spectral features without any sample pretreatment [3–11]. Fourier transform infrared (FTIR) spectroscopy provides a direct assessment of biochemical composition of pollen which has proven to be species specific [7, 8]. Therefore, infrared spectroscopy is developing as an important tool in biology for studying phylogenetic differences between pollen grains of diverse plant species.

While most of the studies conducted with infrared spectroscopy were performed by bulk measurements of pollen samples, single grain measurements by FTIR microspectroscopy on single pollen grains are rare [4]. In addition to FTIR spectroscopy, characterization and classification of pollen grains have been performed by Raman spectroscopy, including detailed microspectroscopy measurements [9, 10, 12]. Although Raman measurements of pollen are often obstructed by the strong fluorescence background and laser-induced degradation, the studies have nevertheless shown a potential of Raman spectroscopy in ecology and aerobiology.

Although several comparative studies employing vibrational spectroscopy have been performed on pollen [5, 8], none of the studies compared the level of chemical information that can be obtained by the different spectroscopic techniques that operate on different scales and can highlight different chemical aspects of pollen. Pollen studies are almost exclusively focused on identification of pollen grains, while the chemical characterization has remained underutilized. However, chemical characterization of pollen could present important information for palaeosciences regarding that some pollen chemicals have long-term stability spanning millions of years [13]. For example, sporopollenin, the main constituent of pollen grain wall, is extremely resilient to a variety of abiotic stresses and well preserved in the fossil record, while its chemical composition depends on environment experienced during the growth of the parent plant [14]. Since phenolic 98i blocks of sporopollenins have characteristic spectral features, vibrational spectroscopy offers a novel approach for chemical analysis of pollen grain wall and improvement of palaeoecological studies [15].

In this study, pollen samples of Pinales (conifer) species were investigated. The measured species are growing in Northern hemisphere and they belong to the most numerous extant group of conifers. The chosen sample set, consisting of 15 different species of Pinaceae and Podocarpaceae families, offers phylogenetic assortment and representation at the family and genus level due to diversity in grain size, shape, and relative biochemical composition. Different vibrational spectroscopic methodologies have been used in a multiscale setting to obtain spectra from the same pollen samples by bulk measurements as well as microscopic measurements at sub-grain level. Application of pollen measurements often requires measurement of an individual pollen grain, such as in monitoring of aeroallergens. Therefore, it is important to assess if the advancement in bulk FTIR measurements of pollen can be repeated at the single grain level by FTIR microspectroscopy, despite the anticipated technical difficulties [3, 16]. Five methodologies were used in FTIR measurements: attenuated total reflectance (ATR) measurements of ground and intact pollen grains, microscopic transmission measurements of multigrain and single pollen grains, and potassium bromide (KBr) pellet measurements of ground pollen. For Raman measurements, two different regions from the same pollen grains were measured, namely saccus and corpus area of bisaccate pollen samples. In total, seven different FTIR and Raman methodologies have been covered, and subsequently combined with multi-block data analysis. The different vibrational methodologies obtain complementary information on pollen substructures, such as grain wall and grain interior, as well on pollen chemical components, such as sporopollenins, proteins, lipids and carotenoids. Multi-block multivariate methods have been used for integrating the different spectroscopic data sets and for extracting and visualizing common underlying patterns of mutual information of the different spectroscopic data blocks, thus enabling interpretation of spectroscopic measurements.

## Materials and Methods

### Samples

15 pollen samples, belonging to 15 different Pinales species, were collected at the Botanical Garden of the Faculty of Science, the University of Zagreb, during pollination season in the spring of 2012 (Table 1). Each pollen sample was collected from one tree, and it includes pollen grains from at least 10 male cones (pollen strobili). The pollen samples were kept at room temperature for 24 hours, and subsequently stored at -15°C in microcentrifuge tubes until measurements time. The study was a part of government-funded research, and has been conducted with the full cooperation of the administrations of the University of Zagreb. The field studies did not involve endangered or protected species.

**Table 1:** List of the pollen samples.

Family	Genus	Species	Name
**Pinaceae**	*Abies*	*A*. *pinsapo*	Spanish Fir
		*A*. *cephalonica*	Greek Fir
	*Cedrus*	*C*. *atlantica*	Atlas Cedar
	*Picea*	*P*. *omorika*	Serbian Spruce
		*P*. *orientalis*	Caucasian Spruce
		*P*. *pungens*	Blue Spruce
	*Pinus*	*P*. *banksiana*	Jack Pine
		*P*. *peuce*	Macedonian Pine
		*P*. *mugo*	Mountain Pine
		*P*. *nigra*	European Black Pine
		*P*. *resinosa*	Red Pine
		*P*. *sylvetris*	Scots Pine
		*P*. *tabuliformis*	Chinese Red Pine
		*P*. *wallichiana*	Himalayan Pine
**Podocarpaceae**	***Podocarpus***	*P*. *neriifolius*	Brown Pine

For chemical characterization of pollen a set of model compounds was measured by FTIR (ATR) and Raman spectroscopies to correlate with high positive or negative values in the principal component analyses correlation plots. Tristearin (1,3-di(octadecanoyloxy)propan-2-yl octadecanoate), triolein (2,3-bis[[(Z)-octadec-9-enoyl]oxy]propyl (Z)-octadec-9-enoate), phosphatidistearoylcholine (1,2-distearoyl-rac-glycero-3-phosphocholine), phosphatidioleylcholine (1,2-dioleoyl-sn-glycero-3-phosphocholine), stearic acid (octadecanoic acid), oleic acid ((9Z)-octadec-9-enoic acid), β-carotene, *p*-coumaric acid, ferulic acid, caffeic acid, sinapic acid, hydro-*p*-coumaric acid, hydroferulic acid, hydrocaffeic acid, cellulose, amylose, amylopectin, arabinoxylan, pectin, β-D-glucan, sucrose, trehalose, fructose, glucose, gluten, α-casein, β-casein, κ-casein were purchased from Merck (Darmstadt, Germany) and Sigma-Aldrich (St. Louis, United States), and used without further purification.

### Spectroscopic analyses

Seven different FTIR and Raman methodologies have been used for the measurement of the same pollen samples. For infrared measurements: reflectance FTIR spectroscopy by Attenuated Total Reflectance (ATR) of ground (1) and intact pollen grains (2); transmission FTIR spectroscopy of KBr pellets (3); transmission FTIR microspectroscopy of multigrain (4) and single pollen grain (5). For Raman microspectroscopy measurements, spectra were obtained from the same pollen grains by focusing on the different substructures: saccus (6) and corpus part (7) of pollen grain.

#### Infrared measurements

Microscopic transmission FTIR measurements (μFTIR) were performed using an Equinox 55 FTIR spectrometer with a IRScopeII IR microscope (Bruker Optik GmbH, Germany). The system is equipped with a globar mid-IR source and a liquid nitrogen-cooled mercury cadmium telluride (MCT) detector. The pollen samples were deposited onto 3 mm thickness of ZnSe optical windows without any sample pretreatment. The spectra were obtained in the 4000–600 cm^-1^ spectral range, with a spectral resolution of 4 cm^-1^, and with 128 scans. Samples were measured using a 15× objective, with different aperture sizes, depending on the size of the sample. The aperture sizes for single pollen grain measurements were ranging from 30xm (for smallest) to 100μm (for biggest) whereas for multigrain pollen samples, fully open aperture (200x200μm) was used. Background (reference) spectra were recorded immediately before starting each measurement by measuring empty areas of 3mm ZnSe slides. Visible images of the measured pollen grains were obtained by a CCD-camera coupled to the microscope. The microscope was equipped with a computer-controlled x, y stage. The Bruker system was controlled with OPUS 6.0 software (Bruker Optik GmbH, Germany).

The reflectance infrared spectra were recorded using an Equinox 55 FTIR spectrometer (Bruker Optik GmbH, Germany) with an ATR accessory. The ATR IR spectra were recorded with a total of 32 scans and spectral resolution of 4 cm^-1^, using the 1.8 mm thickness of horizontal single-reflection ATR diamond prism with 45° angle of incidence on a MIRacle ATR accessory (PIKE Technologies, USA). Background (reference) spectra were recorded immediately before starting each measurement using the sample-free setup. Pollen samples were measured using two different sampling methodologies: intact samples as collected in nature, and samples ground using an agate pestle and mortar. For ground measurement, batch of 5 mg pollen sample was grounded to a fine homogenous powder using an agate pestle and mortar, and roughly 1 mg of sample per measurement was deposited onto ATR crystal (3 replicate measurements were obtained). Data acquisition and instrument control was carried out using the OPUS 6.0 software (Bruker Optik GmbH, Germany).

The transmission spectra of KBr sample pellets were collected with 4 cm^-1^ resolution acquiring a total of 30 scans on an MB102 single beam FTIR spectrometer (ABB Bomem, Canada), equipped with CsI optics and a DTGS detector (Deuterated Triglycine Sulfate). Background (reference) spectra were recorded immediately before starting each measurement using the sample-free setup. KBr pellets were prepared by mixing approx. 1 mg of a sample with approx. 100 mg of KBr and grounded the mixture to a fine homogenous powder mixture using an agate pestle and mortar. Ground powder samples were transferred to the 10 mm pellet die assembly and the KBr matrix was then cold-pressed without degassing into a transparent disk. Three KBr pellets were prepared and recorded for each pollen sample. Data acquisition and instrument control was carried out using the GRAMS/32 software (Galactic Industries Corp., USA)

#### Raman measurements

By Raman microspectroscopy (μRaman), spectra of different regions of pollen grains were obtained by manually focusing on either saccus or corpus region of the grain. Raman spectra were recorded by a LabRam HR 800 Raman microscope (Horiba Scientific, France). The excitation wavelength of 632.8 nm was generated by a He–Ne laser. A 100× objective (Olympus, France) was used for focusing and collecting scattered Raman light. The laser power was approximately 15 mW on the sample surface. The confocal hole was set to 20 μm, with a laser spot diameter of approx. 0.8 μm, a spectral resolution of 2 cm^-1^, and an exposure time of 4 x 10 s. The Raman scattering was dispersed with a 300 lines/mm grating, which resulted in spectra in range 409–2611 cm^-1^. Data acquisition and instrument control was carried out using the LabSpec 5.45 software (Horiba Scientific, France).

#### Scanning electron microscopy

The scanning electron microscope (SEM) images were taken with Zeiss EVO 50 Extended Pressure scanning electron microscope (Carl Zeiss AG, Jena, Germany). The desiccated pollen samples were attached to SEM stubs (covered with double-stick tape) without prior pre-treatment with chemicals. The samples were coated with gold-palladium and measured with the SEM.

### Data analysis

#### Spectral pre-processing

For the analysis of the spectral sets, the spectral region of 1900 to 700 cm^−1^ was selected. This spectral region contains bands that are distinctive for pollen grains [3, 4, 7, 8]. Spectra were smoothed and transformed to second derivative form by the Savitzky-Golay (SG) algorithm using a polynomial of power 2 with different window sizes depending on the spectral data sets (15 for bulk FTIR measurements, 17 for FTIR microspectroscopy, and 25 for Raman measurements). The window sizes were chosen according to the number of channel readings and the noise level in the respective data set [17]. After derivation by the SG algorithm, spectra were processed using extended multiplicative signal correction (EMSC). The SG algorithm was used to enhance spectral features, while the EMSC pre-processing was used for normalization and for separation of chemical and physical variations in vibrational spectra [17, 18].

#### Multiblock principal component analysis

Principal component analysis (PCA) of pre-processed spectra was used to evaluate biochemical differences between different pollen taxa by displaying main variation patterns in score plots for each spectroscopic method. The multiblock method Consensus Principal Component Analysis (CPCA) was used to combine the seven measurement methods by integrating multivariate signals from each technique (i.e. data block) in one data model [19–22]. Following this approach, the relations between the molecular signatures traced by different techniques were obtained, as well as the variation pattern in the whole (multiblock) data set. In order to integrate different data blocks for CPCA, a row-to-row correspondence between the data blocks was obtained. Therefore, the spectra were averaged over all replicates for each spectroscopic method and integrated as seven blocks: Block 1) KBR (transmission FTIR of KBr pellets), block 2) ATI (ATR-FTIR of intact pollen), block 3) ATG (ATR-FTIR of ground pollen), block 4) MGR (transmission μFTIR of multigrain), block 5) SGR (transmission μFTIR of single grain), block 6) RMC (Raman of corpus region) and block 7) RMS (Raman of saccus region).

504 spectra were analysed in total, including at least 3 replicates per sample for each methodology. For bulk measurements (KBR, ATI and ATG), replicates refer to measurements of distinct aliquots of the pollen sample (approx. 1 mg per measurement). For multigrain μFTIR measurements (MGR), replicates refer to measurements of distinct regions on the microscope slide. For single grain measurements (SGR, RMC and RMS), replicates refer to measurements of distinct pollen grains. For MGR and SGR measurements, the number of replicate measurements was between 5 and 10 spectra, and it was not uniform for all species. CPCA was performed as described in literature [19, 23]. In order to illustrate the relations and variation patterns in sample and variable spaces, score and correlation loading plots were used [19, 24, 25]. In order to visualize variables in correlation loading plots and in order to avoid overcrowded plots, spectral band positions were identified by determining minima in second derivative spectra. When band shifts were occurring in a data set, all shifted band positions were used for each spectral band. It is important to note though, that CPCA models were performed on all variables, not only the selected band positions. Thus, all score plots are based on the whole spectral region from 1900 to 700 cm^−1^. All pre-processing methods and data analysis were performed using in-house developed routines written in MATLAB V. 8.3.0.532 (The MathWorks, Natick, USA).

## Results and Discussion

**Fig 1:**
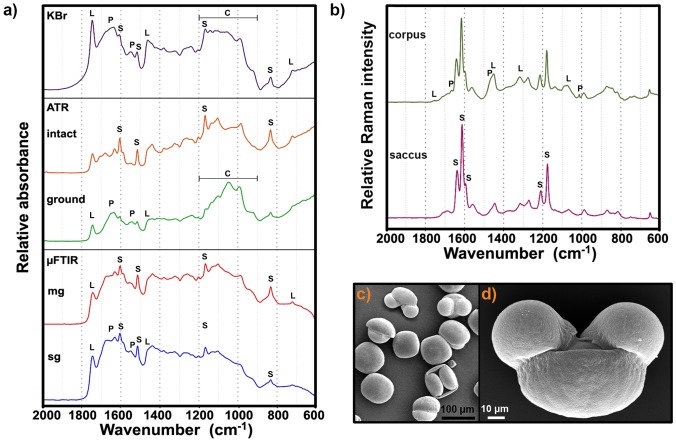
Measurement of *Abies cephalonica* pollen. **(a)** FTIR spectra obtained from different sampling techniques (from top downwards): transmission IR of KBr pellet, ATR-IR of intact and ground grains, IR microspectroscopy of multigrain (mg) and single grain (sg). **(b)** Raman spectra obtained from two different regions of a single grain: corpus and saccus regions. The marked signals are associated with the vibrational bands of (P) proteins, (L) lipids, (C) carbohydrates and (S) sporopollenins. **(c)** SEM image of pollen grains in various orientations: equatorial view (up left), distal polar view (up right) and proximal polar view (down left). **(d)** SEM image of pollen grain in equatorial view, with saccus (up, two hemispherical substructures) and corpus regions (down, large hemispherical substructure).

The sample set covers conifer taxa with characteristic morphological and biochemical properties (Table 1). In general, pollen grains have double layered grain walls, with an inner layer (intine) that is predominantly composed of pectin and cellulose, and an outer layer (exine) that is a complex biopolymer sporopollenin [26]. The grains of Pinaceae and Podocarpaceae families have a distinctive hollow projection called the saccus from the central body of pollen grain called the corpus. While the grain interior has a complex composition of proteins, lipids, carbohydrates and nucleic acids, the saccus region is composed mainly of the exine grain wall. All the pollen grains we measured were bisaccate pollen since they have two sacci. In Fig 1 it can be seen that these two sacci are only visible when the orientation of the pollen grain allows it. The grain sizes of the measured taxa range from 50 μm for the species of *Podocarpus*, 55–75 μm for *Pinus*, 75 μm for *Cedrus*, 85 μm for *Picea*, to 120 μm for *Abies*. The sample set has a well defined phylogenetic relationship of the species, with one clear outlier: The species *Podocarpus neriifolius* belongs to the family Podocarpaceae while the rest of the samples belong to the family Pinaceae. Within the Pinaceae, the set contains one large subset belonging to the clade of sister genera *Pinus* comprising 8 species and *Picea* comprising 3 species [27]. In addition, it contains one smaller subset, with a clade that is far related to the genera *Pinus* and *Picea*, belonging to the clade of the sister genera *Abies* comprising 2 species and *Cedrus* comprising 1 species.

### Infrared measurements

**Fig 2:**
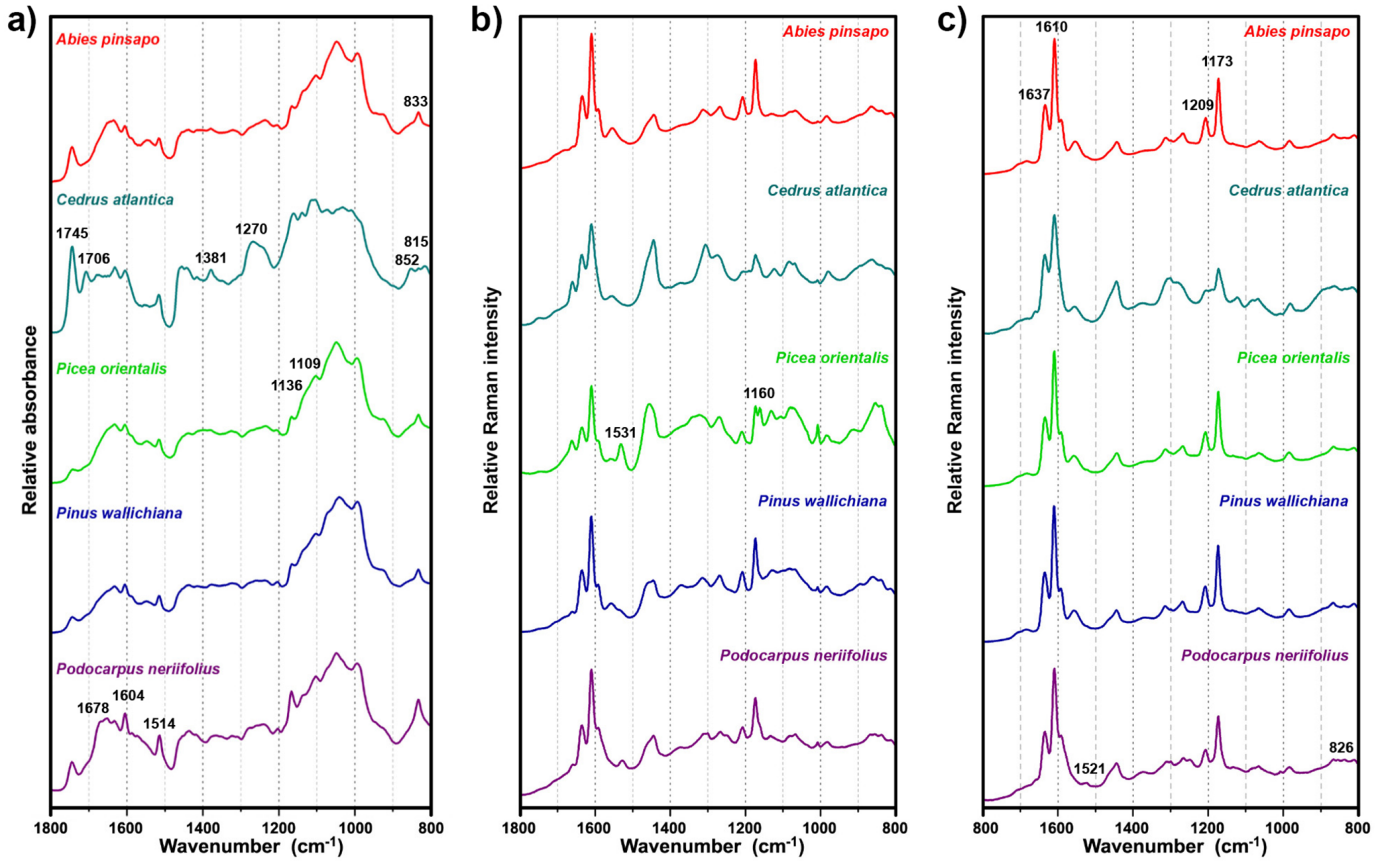
Spectra of representative samples of pollen measured as: (a) ATR of ground pollen, (b) Raman of corpus region, and (c) Raman of saccus region. The spectral set consists of EMSC normalized average spectra; for better viewing the spectra are offset.

A set of representative FTIR spectra, belonging to the same *Abies cephalonica* pollen sample and obtained by different methodologies, are shown in Fig 1. The vibrational spectra of five pollen species are shown in Fig 2. Regarding the amount of the measured pollen samples, the different vibrational techniques comprise bulk (KBr and ATR) and microscopic measurements (μFTIR and μRaman). Regarding the preparation of the pollen samples, the measurements include both ground (KBr and ATR ground) and intact samples (ATR intact, μFTIR and μRaman). Moreover, transmission μFTIR of single grain and multigrain measurements were performed. Finally, different substructures of pollen grains, namely saccus and corpus, were measured by μRaman.

**Fig 3:**
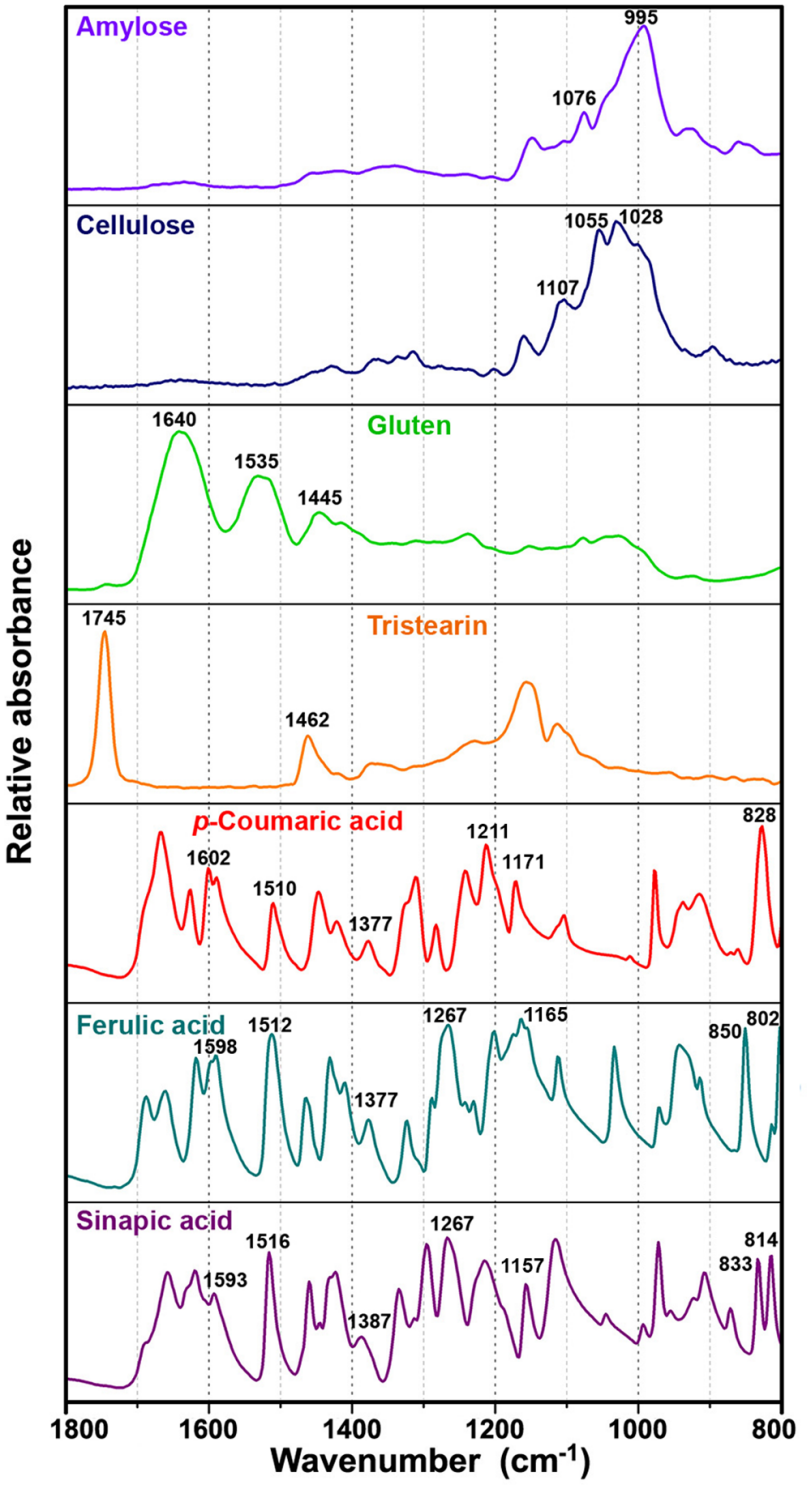
FTIR spectra of biochemicals. carbohydrates (amylose and cellulose), protein (gluten), lipid (tristearin) and phenylpropanoids (*p*-coumaric, ferulic and sinapic acids); for better viewing the spectra are offset.

The FTIR spectra of pollen grains contain information on the major chemical constituents of pollen, such as lipids, proteins, carbohydrates, and grain wall biopolymers sporopollenins and cellulose [4, 5, 8]. The reference infrared spectra of pure compounds that can be typically found in pollen are shown in Fig 3. Lipids are characterized by the strong vibrational band at 1745 cm^-1^ (C = O stretch), as well as by a weaker band at 1462 cm^-1^ (CH_2_ deformation), while proteins are characterized by two strong and broad bands at 1640 cm^-1^ (amide I: C = O stretch) and 1535 cm^-1^ (amide II: NH deformation and C–N stretch). Carbohydrates have strong absorption in the 1200–900 cm^-1^ region (C–O–C and C–OH stretch) including some characteristic bands for certain types of carbohydrates, such as cellulose (at 1107, 1055 and 1028 cm^-1^) and amylose (at 1076 and 995 cm^-1^). Sporopollenins are an extremely resistant group of biopolymers present in wall of pollen and spores. They are complex dehydrogenation-type biopolymers based on phenylpropanoid acids [26]. Therefore, their vibrational spectra show distinctive bands associated with the vibrations of aromatic rings at 1605, 1515, 1171, 853, 833 and 816 cm^-1^ [8]. These bands are present in the spectra of phenylpropanoid acids (Fig 3).

Although all FTIR spectra shown in Fig 1 contain information on the same chemical constituents, it can be seen that the ratio of chemical signals varies substantially from spectrum to spectrum. For instance, there is a considerable difference between the ATR spectra of ground and intact pollen. While the spectrum of intact pollen has predominant signals of sporopollenins, the spectrum of ground grains has predominant signals of lipids and proteins. In contrast, the spectra of KBr pellets and ATR ground samples contain similar information irrespective of the measurement technique. Both spectra are associated with the chemical components from the grain interior (lipids and proteins), rather than grain wall materials (sporopollenins). As opposed to transmission measurement where infrared light interacts with the whole sample, the infrared light measured in reflection measurements penetrates the pollen grains only up to 0.5–5 μm in depth, depending on the wavelength [28]. It means that while in transmission measurements the whole pollen grain is measured, predominantly the grain wall is measured in reflection measurements [7, 8]. Therefore, for large pollen grains, such as Pinaceae and Podocarpaceae covered in this study, a more complete biochemical fingerprint is obtained by grinding the sample.

Regarding FTIR microspectroscopy measurements, it can be seen that both settings (multigrain and single grain) obtain high quality spectra. The measured pollens have quite large sizes, which enables measurement of single grain spectra with high signal-to-noise ratio. For pollen grain that are smaller than 50 μm, the quality of the obtained spectra is often poor due to strong Mie-type scattering effects [16, 29].

The main difference between the obtained multigrain and single grain spectra is their reproducibility. While the reproducibility of multigrain spectra is generally high, the reproducibility of single grain spectra is rather poor. Single grain spectra show high variation in ratio of principal bands belonging to grain interior and grain wall [29]. The reason for this is spatial orientation of a measured grain, which can either favour signals of saccus compounds such as sporopollenins or signals from corpus compounds such as nutrients (see grain orientations in Fig 1c). For instance, a measurement of the grain with equatorial profile orientation results in relatively stronger signals of proteins than sporopollenins, while the reverse is true for the grain with distal polar orientation [29].

It should be noted that spectral differences between pollen samples are related not only to phylogenetic differences between pollen species but to environmental conditions affecting parent plants as well. Pollen samples obtained from the same trees in different years can show noticeable inter-annual variations of biochemical composition. For instance, pollen samples of the specific *Abies cephalonica* tree, sampled in this study, were collected in consecutive pollination seasons, in 2011, 2012 and 2013. The infrared spectra of samples collected in different seasons show noticeable spectral differences associated with ratios of principal chemical constituents. The ATR (intact) FTIR spectra for *Abies cephalonica* in Fig 1a (pollination season 2012) can be compared with the ATR spectra in [30] (pollination season 2011), while the results of inter-annual variations of pollen data for this and other conifer trees was presented in [7]. Therefore, identification of pollen species based on their vibrational spectra is a complex issue that needs to take into consideration variability within species (i.e. variability within population and between different populations), as well as temporal variability within individual mother plant (i.e. variability between different pollination seasons).

### Raman measurements

**Fig 4:**
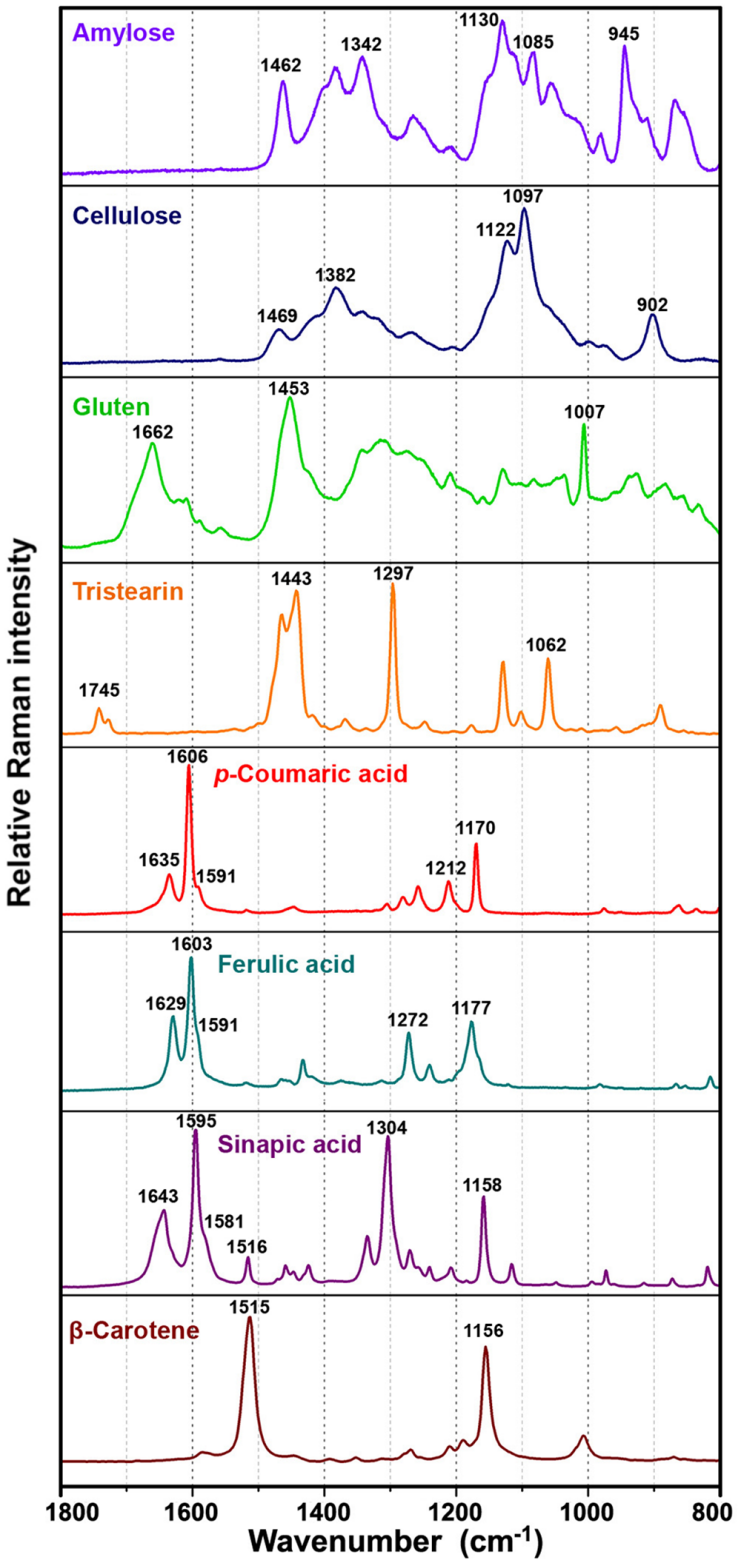
Raman spectra of biochemicals. carbohydrates (amylose and cellulose), protein (gluten), lipid (tristearin), phenylpropanoids (p-coumaric, ferulic and sinapic acids) and carotenoid (β-carotene); for better viewing the spectra are offset.

A set of representative Raman spectra obtained from samples belonging to the same species *Abies cephalonica* are shown in Fig 1. In order to investigate the spectral reproducibility and the chemical heterogeneity of single pollen grains, Raman spectra were collected from different positions on each single pollen grain. As shown in Fig 1, there are distinct differences between spectra obtained from the saccus and corpus structures. The presence of additional signals in the spectrum of the corpus structure is obvious. Spectra obtained from Raman measurements of saccus and corpus region of pollen species are shown in the Supplementary information (Fig J in S1 File). Similar to FTIR spectra of pollen, Raman spectra contain information on the major chemical constituents [6, 8, 31]. The main spectral features, that are dominating both saccus and corpus Raman spectra, are signals at 1637, 1610, 1590, 1209 and 1173 cm^-1^, which can be associated with sporopollenins, that is with phenylpropanoid building blocks. The additional signals, present in the corpus spectrum, can be associated with lipids (1750, 1444, 1304 and 1065 cm^-1^) and proteins (1660, 1455, 1007 cm^-1^). Reference Raman spectra of typical pure constituents of pollen samples are shown in the Fig 4. The results are in agreement with pollen grain anatomy, since the saccus regions are predominantly made of sporopollenins while the corpus region contains nutrients and coding chemicals.

Since pollen grains have a layered and chemically inhomogeneous structure, the acquired spectra differ when the depth of the laser focus is changed. For saccate pollen, the inhomogeneity is not only due to a layered structure, but also due to the fact that the grain has a saccus and a corpus region which represent two completely different substructures. Since the saccus region is a pretty homogeneous region, Raman measurements of saccus substructures result in a visually pure spectrum of sporopollenins. All principal bands can be associated with phenylpropanoid building blocks of sporopollenins, as indicated by the similarity between the Raman spectrum of saccus region (Fig 1b) and the spectra of phenylpropanoid acids (Fig 4). Raman measurements of the corpus region may result in varying signals of lipids and proteins strongly depending on the focus depth of the laser. Therefore, in contrast to findings of a previous Raman study [32], it is challenging to obtain reproducible Raman spectra of single pollen grains.

### Consensus principal component analysis (CPCA)

The spectral data sets were pre-processed prior to CPCA in order to remove baseline variations and differences in the effective optical path length and in order to enhance the spectral signal [33]. These unwanted variations arise due to non-ideal instrument and sample properties, such as fluorescence backgrounds in Raman and variability between KBr sample pellets in FTIR due to slight differences in sample concentration. The spectra were smoothed and transformed to second derivative form by the SG algorithm followed by EMSC pre-processing. Second derivative spectra belonging to the different vibrational methodologies are given in the Supplementary information (Figs A–G in S1 File). In order to estimate variability within sample replicates, spectra from *Pinus* and *Picea* genera were used (Table A and Table B in S1 File). The variability within replicates shows that the highest reproducibility was obtained by infrared measurements on bulk samples (ATI), as well as by Raman microspectroscopy of measurements of corpus region of pollen grain (RMC). Moreover, the variability between KBR sample pellets (KBR), as well as between differently orientated single pollen grains (SGR), results with relatively low reproducibility.

**Fig 5:**
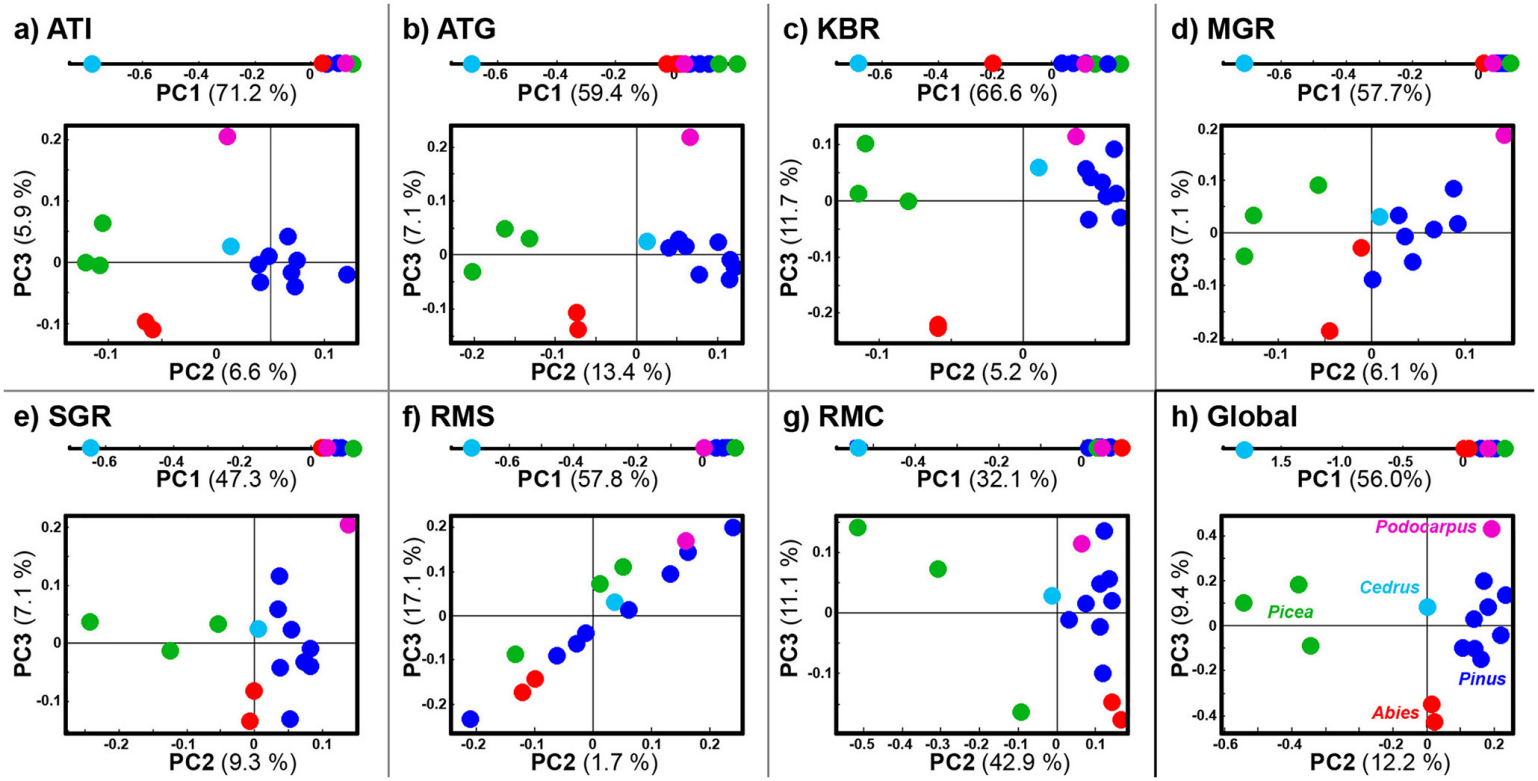
Score plots of individual blocks and global scores of consensus principal component analysis (CPCA). ATR-FTIR of **(a)** (ATI) intact pollen, **(b)** (ATG) ground pollen; **(c)** (KBR) Transmission FTIR of KBr pellets; Transmission IR microspectroscopy of **(d)** (MGR) multigrain and **(e)** (SGR) single grain; Raman spectroscopy of **(f)** (RMS) saccus region and **(g)** (RMC) corpus region of single pollen grain; **(h)** Global scores. Samples are labelled in accordance to pollen genus: Abies (red), Picea (green), Pinus (blue), Podocarpus (magenta), and Cedrus (cyan). The percent variances for the PCs are given in supplementary part.

**Fig 6:**
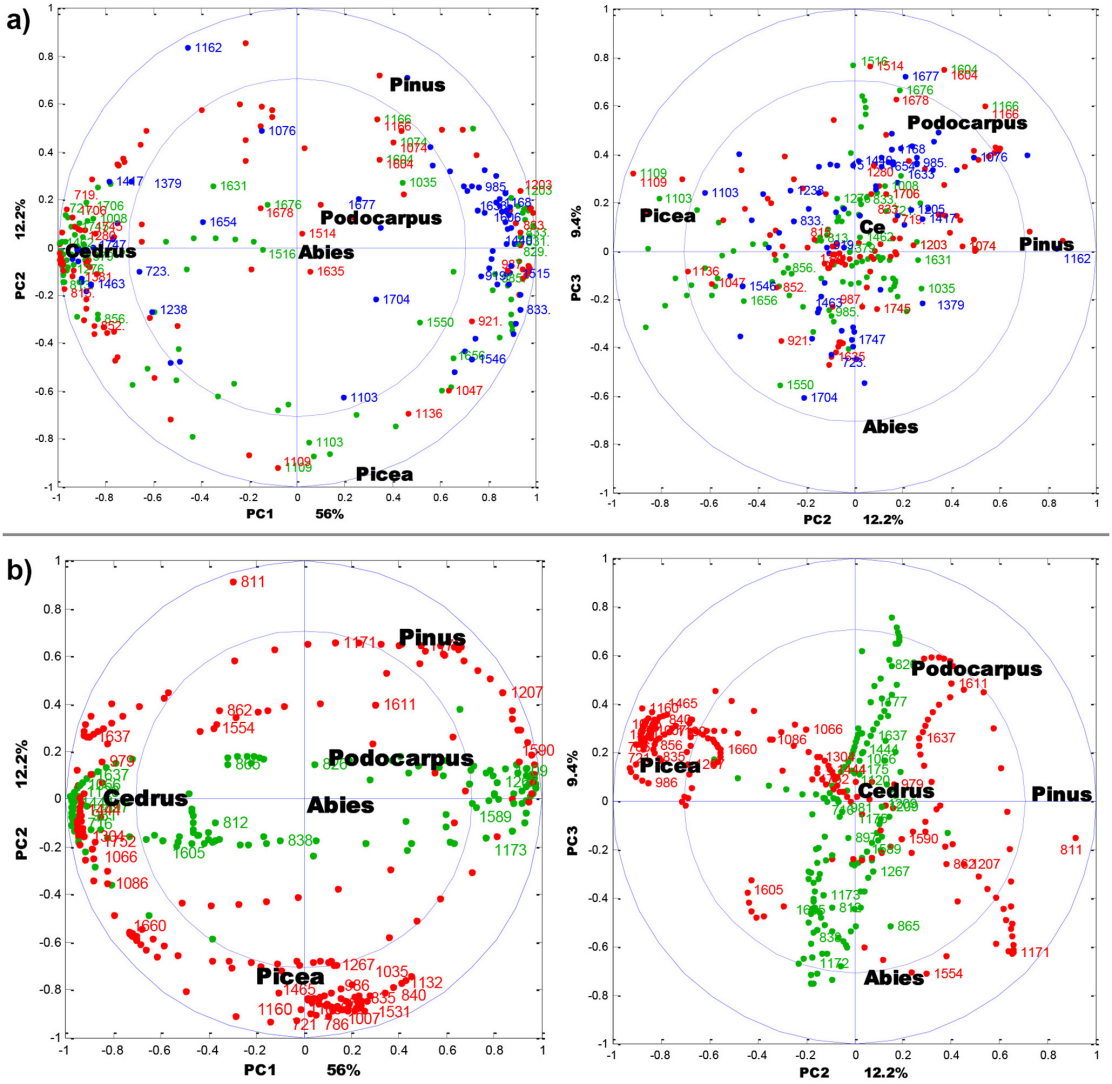
CPCA correlation loading plots for the first three principal components. **(a)** Correlation between pollen genera (black), FTIR of KBr pellets (blue), ATR of ground pollen (red) and ATR of intact pollen (green). **(b)** Correlation between pollen genera (black), Raman of corpus region (red) and Raman of saccus region (green). For the sake of clarity, only the selected variables are presented. The percent variances for the first five PCs are 56.0, 12.2, 9.4, 7.5 and 5.1.

The seven spectral data blocks were analysed by multiblock analysis (CPCA) in order to assess and compare the power of different vibrational methodologies for the analysis of different chemical aspects of pollen. The strength of a multiblock analysis compared to principal component analysis of each block separately is, that analytical aspects that are common and particular for the different spectroscopic methods can be revealed more straight-forward in CPCA. Further, spectral bands that are important for observed sample variation patterns in the different blocks can be visualized in a common multiblock correlation loading block. By CPCA so-called global and block scores are obtained. The global scores of the CPCA are representing the consensus variation between all vibrational methods, while the block scores are representing the consensus variation within each single block and are thus reflecting block-specific variation patterns (Fig 5). The fact that CPCA finds a common variation pattern for all blocks in each component, constitutes the major difference to a PCA analysis of each single block, separately: In PCA of separate blocks, the results obtained for each component are not comparable among the blocks. Therefore, by assessing global and block scores in CPCA, direct information about similarities and differences between the different vibrational methods can be obtained. The high fraction of explained variance for a single block indicates that the global pattern of the CPCA is providing good explanation for the variation within that block, and vice versa. In correlation loading plots the correlations between global scores (principal components) and vibrational bands are plotted [19, 23] (Fig 6). This allows the visualization of correlations between vibrational bands and principal components for all methods in one picture (blocks). Further, by including information about pollen genera in the correlation loading plots, correlations between vibrational bands and genus information can be studied (Fig 6). This is achieved by defining a matrix of indicator variables with zeros and ones that specifies if a sample belongs to a genus or not [23]. Variables that are well explained by respective components in CPCA are located within the outer ring, where the outer circle indicated complete correlation and the inner circle indicates 50% correlation. In addition to Figs 5 and 6, more detailed results are presented in the Supplementary Information (Figs A–G in S1 File). 3D presentations of global score plot are given in the SFig H in S1 File, alongside PCA score plots of individuals blocks (Fig I in S1 File). The score plots, obtained by a separate PCA of each individual block are consistent with the CPCA results, and thus will not be discussed further in this study. Spectra of the same samples and the PCA score plots of individual blocks (with designation at species level), show good reproducibility of spectral measurements (Fig J and Fig K in S1 File).

The consensus variation pattern that was obtained in the first three components of the global score plot reflects the differences between the five different pollen genera as shown in Fig 5h. While PC1 is clearly separating *Cedrus* from the other genera, PC2 is separating *Picea* and *Pinus* and PC3 is separating *Abies* and *Podocarpus*. Therefore, the first three PCs are sufficient for separating the five Pinales genera. Similar grouping patterns as revealed by the global scores (Fig 5h) are found in the block score plots of the ATR block of intact pollen samples (Fig 5a), of the ATR block of ground pollen samples (Fig 5b), and in the block score plot of the KBr pellet block (Fig 5c). The microspectroscopy measurements, both Raman and FTIR, reveal different patterns (Fig 5d–5g). While the first principal components are as before separating *Cedrus* from the other genera, PC2 and PC3 scores are not providing a clear separation of pollen genera. Especially the Raman spectra obtained from the saccus region do not show any grouping with respect to genera, which is also reflected in the low explained variances in PC2 (Fig 5f).

### Biochemical composition of pollen

The correlation loadings for the CPCA model are shown in Fig 6, and additional information with more detailed correlation loading plots and spectral data are presented in the Supplementary Information (Figs A–G in S1 File). Infrared bands associated with sporopollenins, lipids and proteins are highly positively or negatively correlated to *Cedrus* for PC1. More specific: In the ATG spectral block, strong positive correlation to *Cedrus* are observed for bands at 1381, 1270, 852 and 815 cm^-1^ referring to sporopollenin and at 1745, 1706 and 720 cm^-1^ referring to lipids. In the ATI spectral block, strong positive correlation to *Cedrus* are observed at bands at 1516, 1270, 856 and 813 cm^-1^ referring to sporopollenin, at 1745, 1462, and 721 cm^-1^ referring to lipids and at 1631 and 1550 cm^-1^ referring to proteins. In the KBR spectral block strong positive correlation to *Cedrus* are observed at bands at 1747, 1463, 1238 and 723 cm^-1^ (lipids) (Fig 6a). In addition, *Cedrus* has a strong negative correlation to the band at 833 cm^-1^ (sporopollenin) in the ATG block, to the bands 1604 and 833 cm^-1^ (sporopollenin) in the ATI block, and to the bands at 1606, 1515 and 833 cm^-1^ (sporopollenin) and to the bands at 1633 and 1546 cm^-1^ (proteins) in the KBR block (Fig 6a). These results show that there are clear and interpretable chemical differences in the biochemical composition between the *Cedrus* pollen and the rest of Pinales pollens.

The relative intensities of lipid and protein bands indicate that *Cedrus atlantica* has significantly higher relative amount of lipids than average Pinales pollen. Even more interesting is the positive and negative correlation between *C*. *atlantica* and a number of sporopollenins bands. Although the exact chemical composition of sporopollenins has yet to be determined, it is known that that these complex biopolymers are composed of phenylpropanoid building blocks [26]. The phenylpropanoid building blocks, primarily derivatives of *p*-coumaric and ferulic acids, have characteristic vibrations, and thus can be determined by FTIR and Raman spectroscopy [34]., The vibrational spectra of phenylpropanoids are shown in Figs 3 and 4. The relative intensities of Raman and infrared bands indicate that *C*. *atlantica* pollen have a higher ratio of ferulic-to-*p*-coumaric acid derivatives in sporopollenin compared to other Pinales pollens. Specifically, this is revealed by the positive correlation between *C*. *atlantica* and the infrared bands of ferulic acid at 1267 cm^-1^ (alkyl rocking vibration), and at 852 and 815 cm^-1^ (ring vibrations), as well as by negative correlation with the bands of *p*-coumaric acid at 833 cm^-1^ (ring vibrations) present in the ATG block (Figs 6a and 2a). The Raman spectrum of *C*. *atlantica* pollen corroborates this notion by a higher ratio of ring stretches doublets (ratio of peaks at 1637 and 1610 cm^-1^), as well as lower ratios of ring bending peaks at 1209 and 1173 cm^-1^ (Fig 2c). The strong positive correlation between *C*. *atlantica* and the band at 1637 cm^-1^, and strong negative correlation to the bands at 1209 and 1173 cm^-1^ can be seen in the RMS block (Fig 6b). The band at 1209 cm^-1^ is characteristic for *p*-coumaric acid, while ferulic acid has a higher ratio of ring stretches doublets than *p*-coumaric acid. Therefore the Raman spectra are fully supporting the notion obtained by FTIR.

Sporopollenins have variable composition which is dependent on species specific pathways for the biosynthesis of sporopollenin from simple phenylpropanoids, such as cinnamic acid. Moreover, phenylpropanoids play important role in mitigating damage caused by UV-B radiation, and a variety of plant species are responding to increased UV-B exposure by increasing the concentrations of these compounds [35]. Since the concentration of phenylpropanoids is correlated with UV-B radiation, and since sporopollenins are extremely resilient to deterioration, quantitative measurement of these compounds in pollen microfossils can be used as UV-B proxy to track changes in the flux of UV-B radiation over geological time [15].

*C*. *atlantica* has survived the last glacial in multiple refuge areas along the Atlas Mountains [36]. Following the glacial, it has spread rapidly along the major mountain chains of northern Algeria and Morocco. *C*. *atlantica* is unique among other measured species by pollination season. *Cedrus* species shed pollen in autumn, a considerable difference to other Pinaceae that pollinate during spring. Pollen germination and rapid tube growth demand excessive energy reserves, particularly during pollination in colder conditions that is characteristic for *C*. *atlantica*. Moreover, the natural habitat of *C*. *atlantica* is at lower latitudes and higher altitudes than habitats of other measured Pinaceae species, both of which is connected with high exposure to UV-B radiation. Therefore, it is highly plausible that pollen traits of *C*. *atlantica*, in the form of high nutrient content and specific sporopollenins composition, are playing an important role in adaptation to extreme mountainous conditions of south Mediterranean.

The separation between *Pinus* and *Picea* genera, present in PC2, is predominantly based on different carbohydrate composition. This is indicated by the strong positive correlation between *Picea* and carbohydrate bands at 1136 and 1109 cm^-1^ in the ATG block, and at 1132 and 1035 cm^-1^ in the RMC block (Fig 6). Moreover, *Picea* genera have strong positive correlation with bands specific for carotenoids at 1531 and 1160 cm^-1^ in the RMC block (Fig 6b). The two bands are associated with C-C single bond (1160 cm^-1^) and double bond stretch (1531 cm^-1^) of the polyene chain (Fig 4) [37]. An important feature of Raman technique is that carotenoids can be measured in complex matrices, such as pollen, by obtaining their Raman spectra under resonant excitation [38]. Carotenoids have an allowed π-π* electronic transition which occurs in the visible region and which gives rise to their strong colours. Resonance Raman spectrum of carotenoids is obtained when the wavelength of the incident laser coincides with electronic transition causing strong enhancement of vibrational bands, particularly those at 1531 and 1160 cm^-1^ that have strong electron-phonon coupling. Even though pollen carotenoids are present at too low concentration to be observed by FTIR, they can be analysed by Raman due to resonant effect.

Compared with other Pinaceae pollens, pollen of *Picea* has significantly higher concentration of carotenoids. Similar to phenylpropanoids, pollen carotenoids are playing important role in photo protection from environmental stress by quenching free radicals [39]. Pinaceae pollen are anemophilous, and therefore photo protection is of outmost importance for survival if airborne grains are carried to higher layers of atmosphere. Studies have shown that pollination distances in Pinaceae can be tens of kilometres, and possibly even greater [40].

The separation between *Podocarpus neriifolius* and *Abies* genera, present in PC3, is based on sporopollenin composition as well. This is indicated by the positive correlation between *P*. *neriifolius* and sporopollenin bands at 1678, 1604 and 1514 cm^-1^ in the ATG block, at 1677 cm^-1^ in the ATI and KBR blocks, at 1611 cm^-1^ in the RMC block, and at 1521 and 826 cm^-1^ in the RMS block (Fig 6). Analogous to *C*. *atlantica*, the results indicate that the chemical composition of sporopollenin in *P*. *neriifolius*, specifically the ratio of phenylpropanoid building blocks, is different from composition in Pinaceae. Unfortunately, the listed spectral differences are not specific and conclusive enough. However they suggest that *P*. *neriifolius* sporopollenin could contain some minor phenylpropanoid building blocks, in addition to *p*-coumaric and ferulic acids. For instance, presence of species-specific Raman bands at 1521 and 1300 cm^-1^ is consistent with presence of sinapic acid (Figs 4 and 6).

The explained variances of CPCA indicate the advantages of different spectroscopic methodologies. The ATI and the RMS blocks are contributing most to the first principal component, regarding FTIR and Raman respectively. These two methodologies are focused on obtaining information associated with pollen grain wall. Therefore, they are best suited for obtaining variations between taxa with different chemical composition of sporopollenins, such is the case between *Cedrus* and the rest of Pinaceae pollens. In the same way, the ATG and the RMC blocks are contributing most to the second principal component, regarding FTIR and Raman respectively. Both of the methodologies are optimized for gathering information on chemical composition of pollen grain interior. For that reason, they are obtaining variations in carbohydrate composition, such is the case between *Picea* and *Pinus* genera.

## Conclusions

The study demonstrated that the vibrational methodologies have great potential for systematic collection of data on ecosystems since phylogenetic variation is well represented in the biochemical composition of pollen. The differences can be clearly connected to specific chemical composition, such as nutrient storage or grain wall biopolymers. For example, the extensive spectral differences between pollen of *Cedrus atlantica* and the rest of Pinaceae family were unambiguously connected with molecular composition, revealing the unique composition of *C*. *atlantica*’s sporopollenin.

Out of the seven tested methodologies, the best taxonomy based differentiation of the pollen was obtained by reflectance (ATR) and transmission (KBr pelleting) IR measurements on bulk samples, as well as by Raman microspectroscopy measurements that are focused on the corpus region of pollen grains. Raman microspectroscopy measurements indicate that measurement area, as well as the depth of focus, can have crucial influence on the obtained data. The FTIR microspectroscopy methods have underperformed, compared to other FTIR methodologies. The predominant reason for this is low reproducibility and insufficient specificity of FTIR microspectroscopy data, caused by variations in spatial orientation of a measured grains and by scattering effects [16, 29]. Therefore, further studies are needed to in order to develop better experimental setting for FTIR microspectroscopy of individual pollen grains.

## Supporting Information

S1 File Contains the following files. **Fig A** CPCA correlation loading plots with markings related to ATI. **Fig B** CPCA correlation loading plots with markings related to ATG. **Fig C** CPCA correlation loading plots with markings related to KBR. **Fig D** CPCA correlation loading plots with markings related to MGR. **Fig E** CPCA correlation loading plots with markings related to SGR. **Fig F** CPCA correlation loading plots with markings related to RMS. **Fig G** CPCA correlation loading plots with markings related to RMC. **Fig H** 3D presentation of global score plot. **Fig I** PCA score plots for individual blocks, genus level. **Fig J** Raman spectra of corpus and saccus region of species. **Fig K** PCA score plots for individual blocks, species level. **Fig L** PCA score plots for ATI block, species level. **Fig M** PCA score plots for KBR block, species level. **Fig N** PCA score plots for RMC block, species level. **Table A** Variability measurements within replicates of *Pinus* genus. **Table B** Variability measurements within replicates of *Picea* genus.
(PDF)
