# Supplementary material for: A Multiscale Vibrational Spectroscopic Approach for Identification and Biochemical Characterization of Pollen
Source: PLoS One. 2015 Sep 16;10(9):e0137899. doi: 10.1371/journal.pone.0137899 (PMC4574200; doi:10.1371/journal.pone.0137899)
Supplement: S1 File — Fig A CPCA correlation loading plots with markings related to ATI. Fig B CPCA correlation loading plots with markings related to ATG. Fig C CPCA correlation loading plots with markings related to KBR. Fig D CPCA correlation loading plots with markings related to MGR. Fig E CPCA correlation loading plots with markings related to SGR. Fig F CPCA correlation loading plots with markings related to RMS. Fig G CPCA correlation loading plots with markings related to RMC. Fig H 3D presentation of global score plot. Fig I PCA score plots for individual blocks, genus level. Fig J Raman spectra of corpus and saccus region of species. Fig K PCA score plots for individual blocks, species level. Fig L PCA score plots for ATI block, species level. Fig M PCA score plots for KBR block, species level. Fig N PCA score plots for RMC block, species level. Table A Variability measurements within replicates of Pinus genus. Table B Variability measurements within replicates of Picea genus. (PDF) [file pone.0137899.s001.pdf]

Supporting Information

A multiscale vibrational spectroscopic approach for identification and biochemical characterization of pollen

Murat Bağcıoğlu[1*], Boris Zimmermann[1], Achim Kohler[1,2]

[1]Department of Mathematical Sciences and Technology, Faculty of Environmental Science and Technology, Norwegian University of Life Sciences, Ås, Norway

[2]Nofima AS, Ås, Norway

*** Corresponding author**

E-mail: murat.bagcioglu@nmbu.no (MB)

Table of Contents **Page**

Fig. A CPCA correlation loading plots with markings related to ATI 2

Fig. B CPCA correlation loading plots with markings related to ATG 5

Fig. C CPCA correlation loading plots with markings related to KBR 8

Fig. D CPCA correlation loading plots with markings related to MGR 11

Fig. E CPCA correlation loading plots with markings related to SGR 14

Fig. F CPCA correlation loading plots with markings related to RMS 17

Fig. G CPCA correlation loading plots with markings related to RMC 20

Fig. H 3D presentation of global score plot 23

Fig. I PCA score plots for individual blocks, genus level 24

Fig. J Raman spectra of corpus and saccus region of species 25

Fig. K PCA score plots for individual blocks, species level 26

Fig. L PCA score plots for ATI block, species level 27

Fig. M PCA score plots for KBR block, species level 28

Fig. N PCA score plots for RMC block, species level 29

Table A Variability measurements within replicates of *Pinus* genus 30

Table B Variability measurements within replicates of *Picea* genus 31

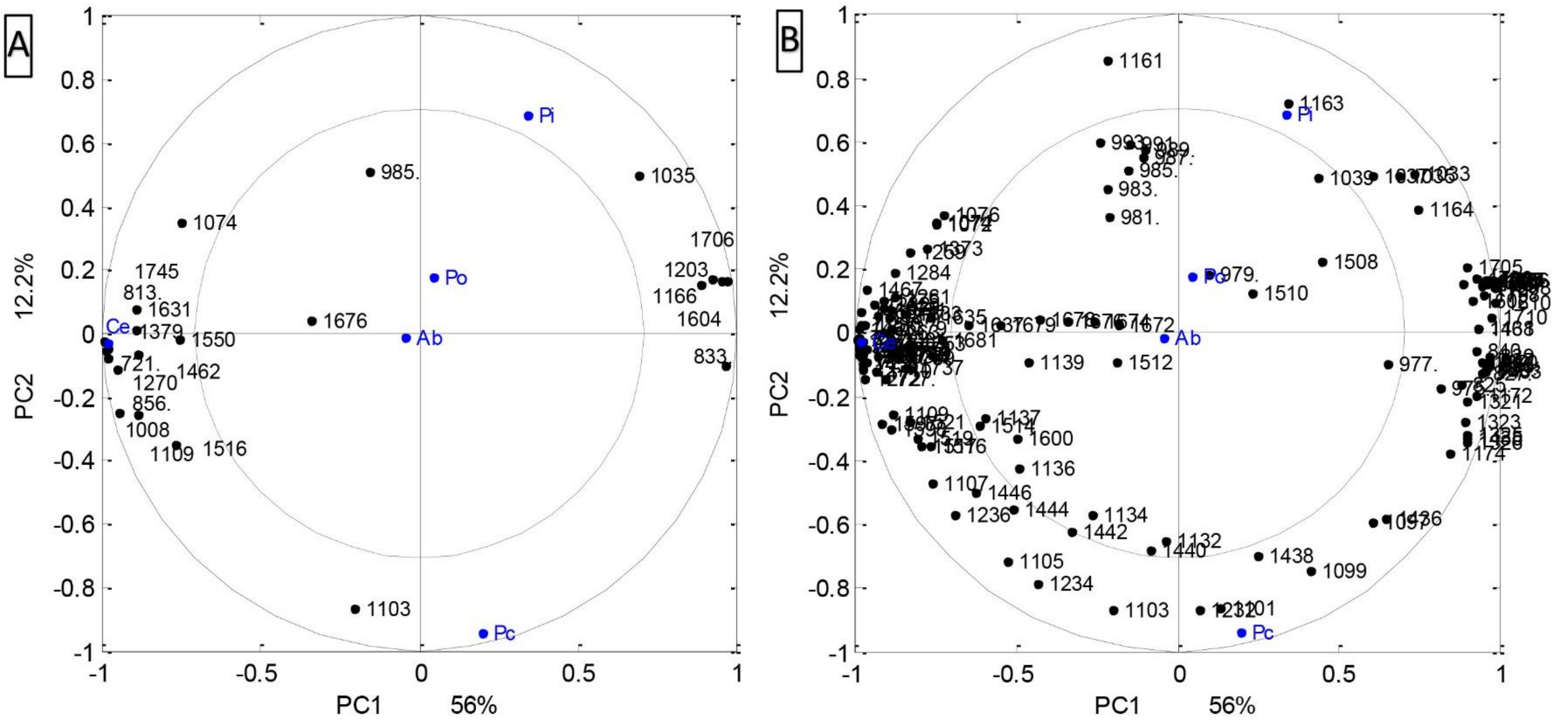

Fig. A (Part 1) CPCA correlation loading plots for the first two principal components. Variables related to ATR IR measurements of intact pollen grains (**ATI**) are presented in black color; **(A)** plot for the selected variables, and **(B)** plot for all variables. Design variables related to plant genera are presented in blue color: *Abies* (Ab), *Cedrus* (Ce), *Picea* (Pc), *Pinus* (Pi), *Podocarpus* (Po).

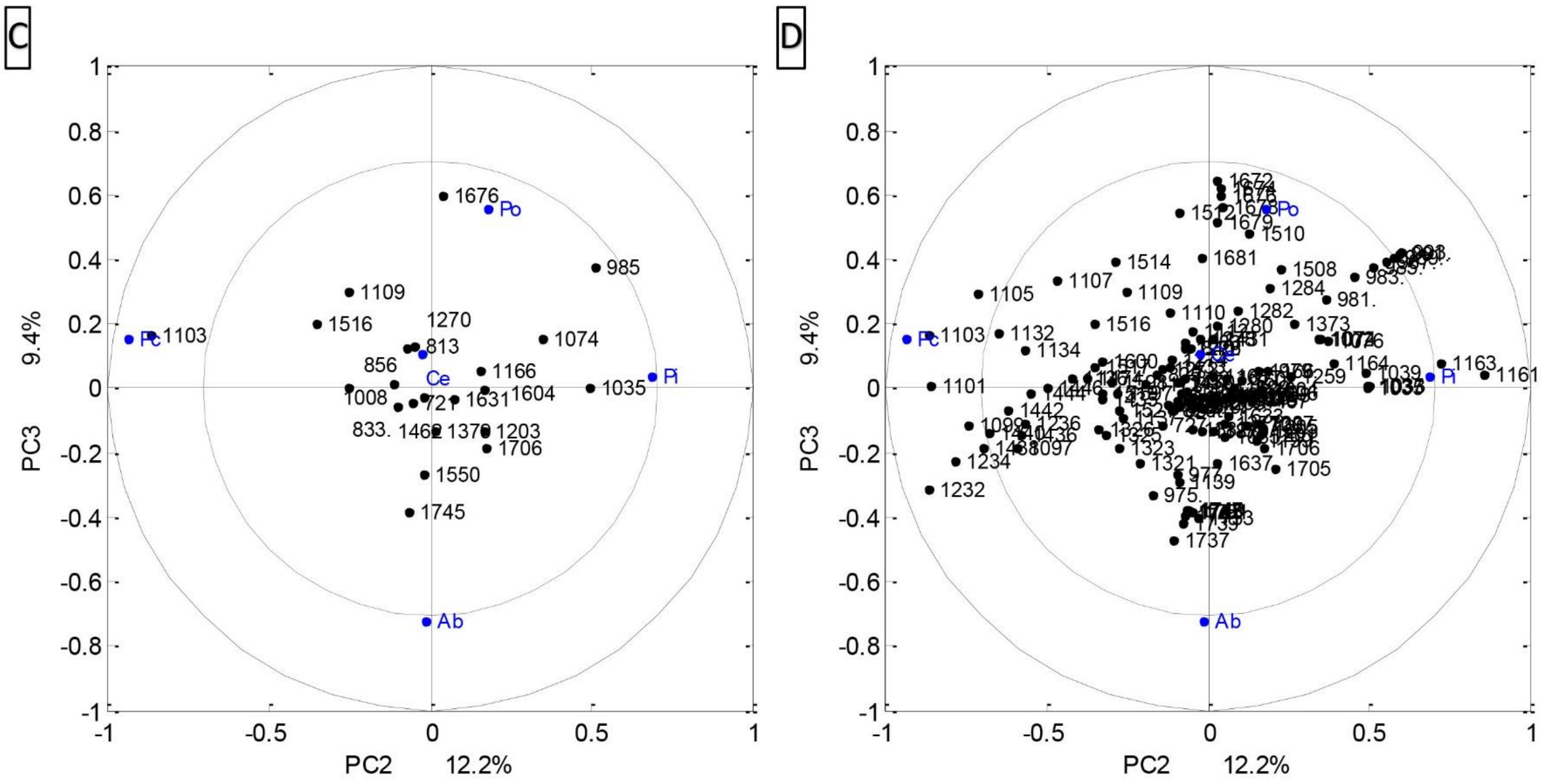

Fig. A (Part 2) CPCA correlation loading plots for the second and the third principal components. Variables related to ATR IR measurements of intact pollen grains (**ATI**) are presented in black color; **(C)** plot for the selected variables, and **(D)** plot for all variables. Design variables related to plant genera are presented in blue color: *Abies* (Ab), *Cedrus* (Ce), *Picea* (Pc), *Pinus* (Pi), *Podocarpus* (Po).

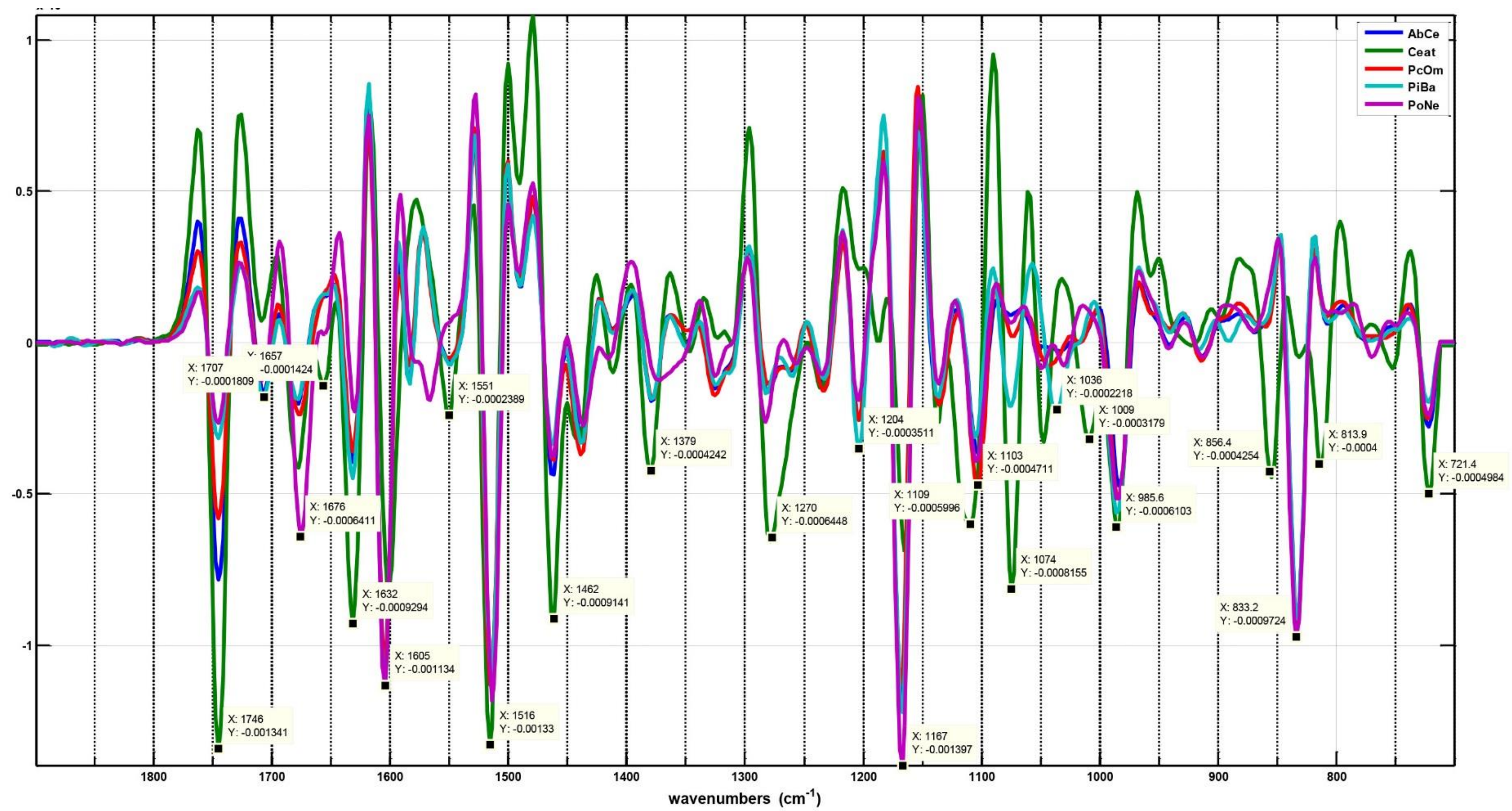

Fig. A (Part 3) Second-derivative and EMSC corrected spectra obtained by ATR IR measurements of intact pollen grains (**ATI**). Average spectra of five representative species are presented: *Abies cephalonica* (AbCe), *Cedrus atlantica* (CeAt), *Picea omorika* (PcOm), *Pinus banksiana* (PiBa), *Podocarpus neriifolius* (PoNe). The selected vibrational bands associated to the CPCA correlation loading plots on Figures S1 are marked.

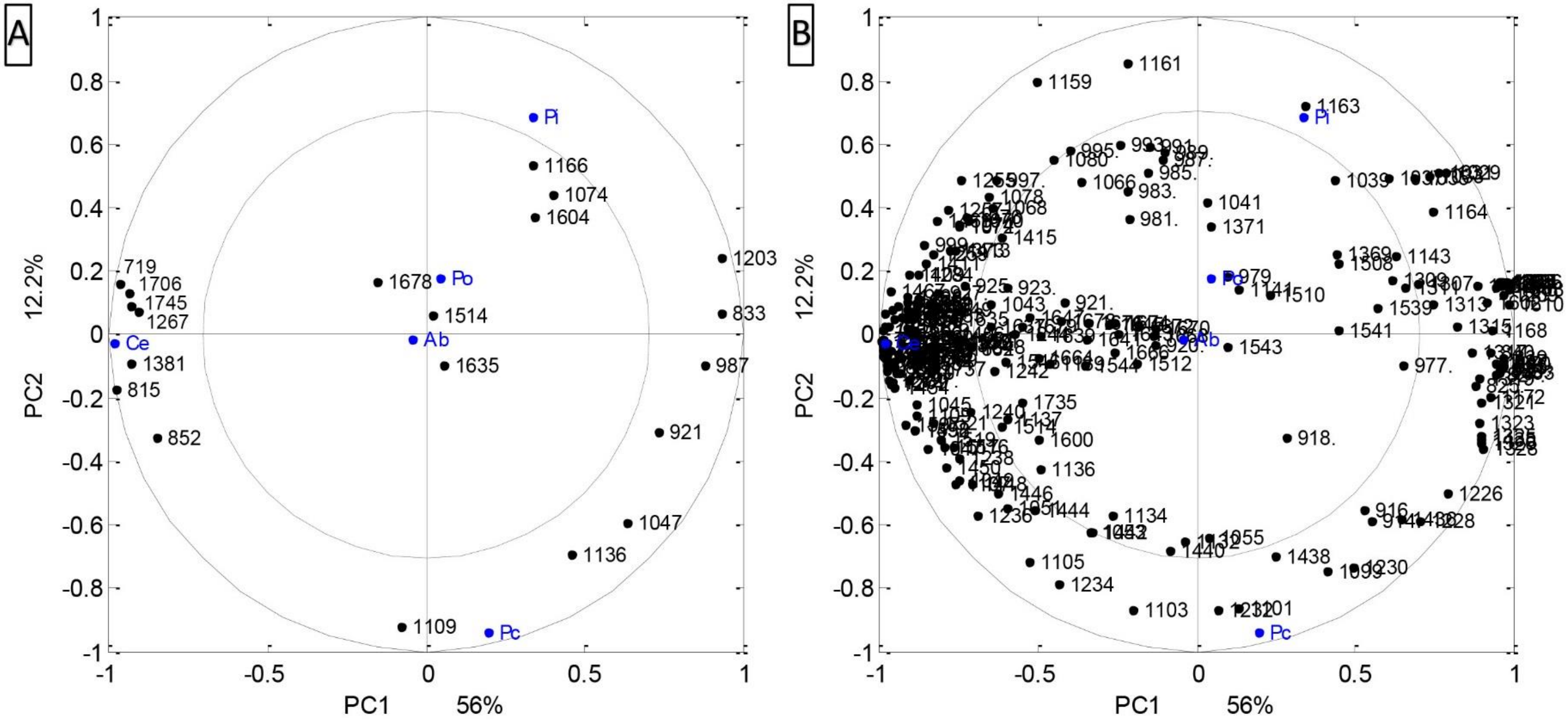

Fig. B (Part 1) CPCA correlation loading plots for the first two principal components. Variables related to ATR IR measurements of ground pollen grains (**ATG**) are presented in black color; **(A)** plot for the selected variables, and **(B)** plot for all variables. Design variables related to plant genera are presented in blue color: *Abies* (Ab), *Cedrus* (Ce), *Picea* (Pc), *Pinus* (Pi), *Podocarpus* (Po).

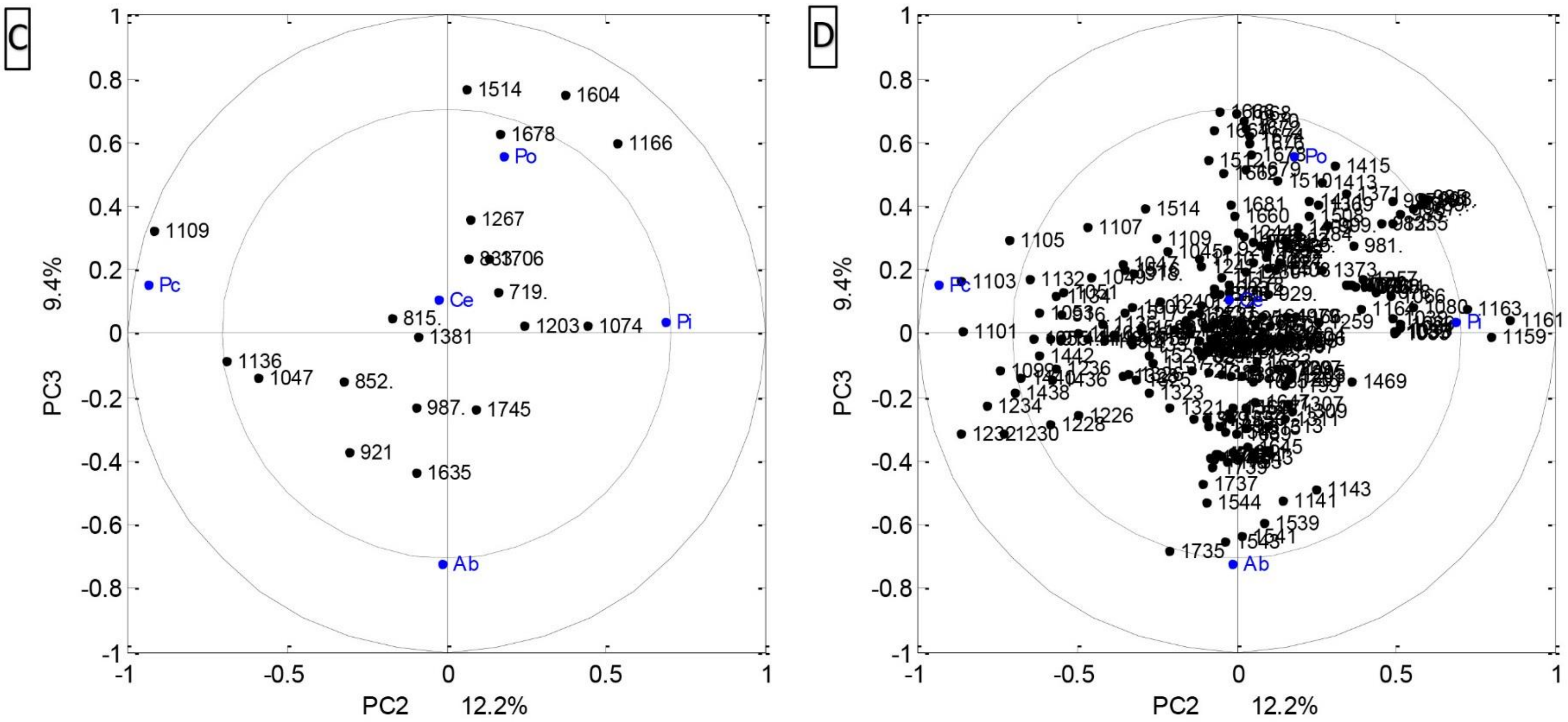

Fig. B (Part 2) CPCA correlation loading plots for the second and the third principal components. Variables related to ATR IR measurements of ground pollen grains (**ATG**) are presented in black color; **(C)** plot for the selected variables, and **(D)** plot for all variables. Design variables related to plant genera are presented in blue color: *Abies* (Ab), *Cedrus* (Ce), *Picea* (Pc), *Pinus* (Pi), *Podocarpus* (Po).

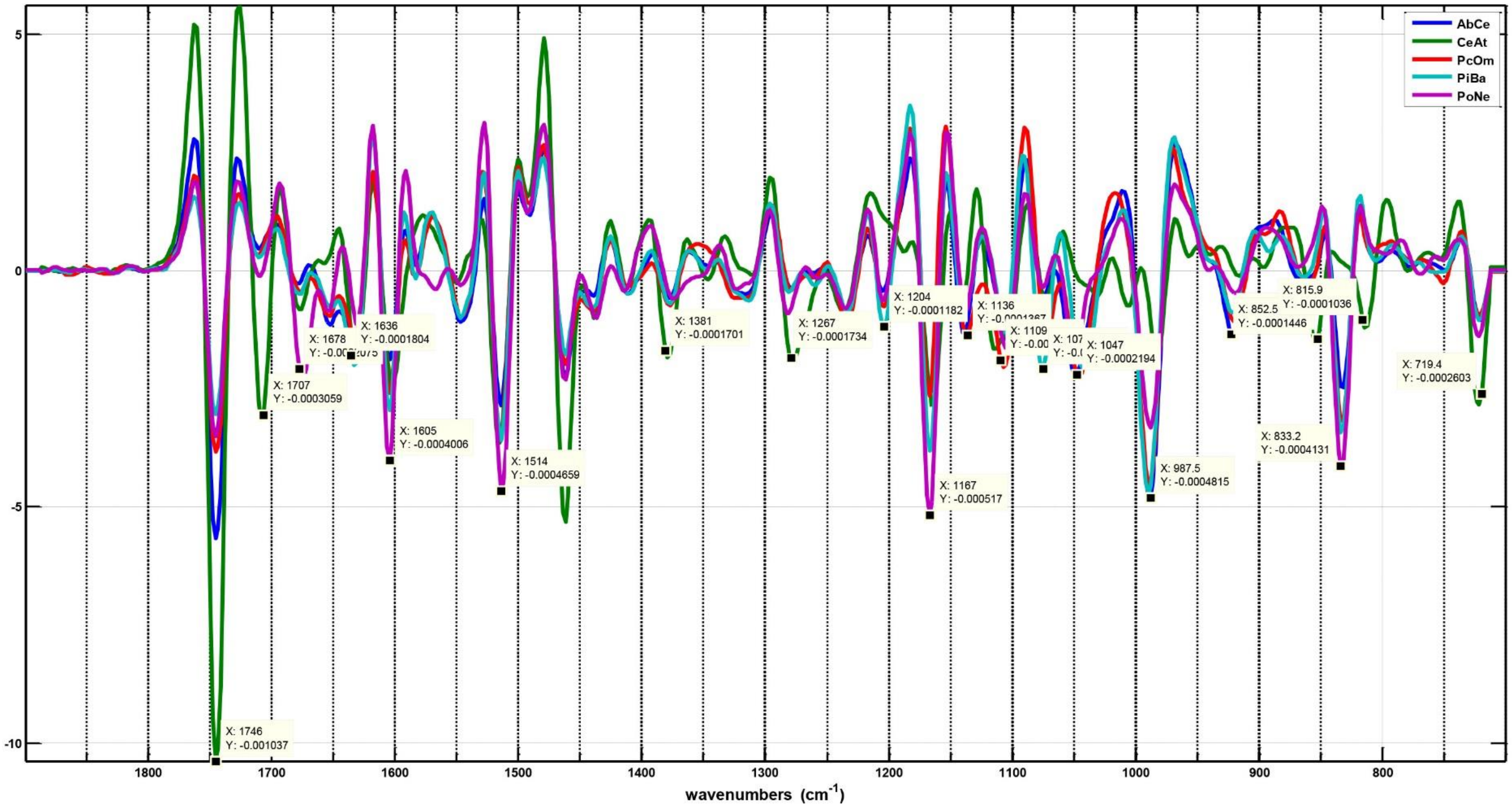

Fig. B (Part 3) Second-derivative and EMSC corrected spectra obtained by ATR IR measurements of ground pollen grains (**ATG**). Average spectra of five representative species are presented: *Abies cephalonica* (AbCe), *Cedrus atlantica* (CeAt), *Picea omorika* (PcOm), *Pinus banksiana* (PiBa), *Podocarpus neriifolius* (PoNe). The selected vibrational bands associated to the CPCA correlation loading plots on Figures S-2 are marked.

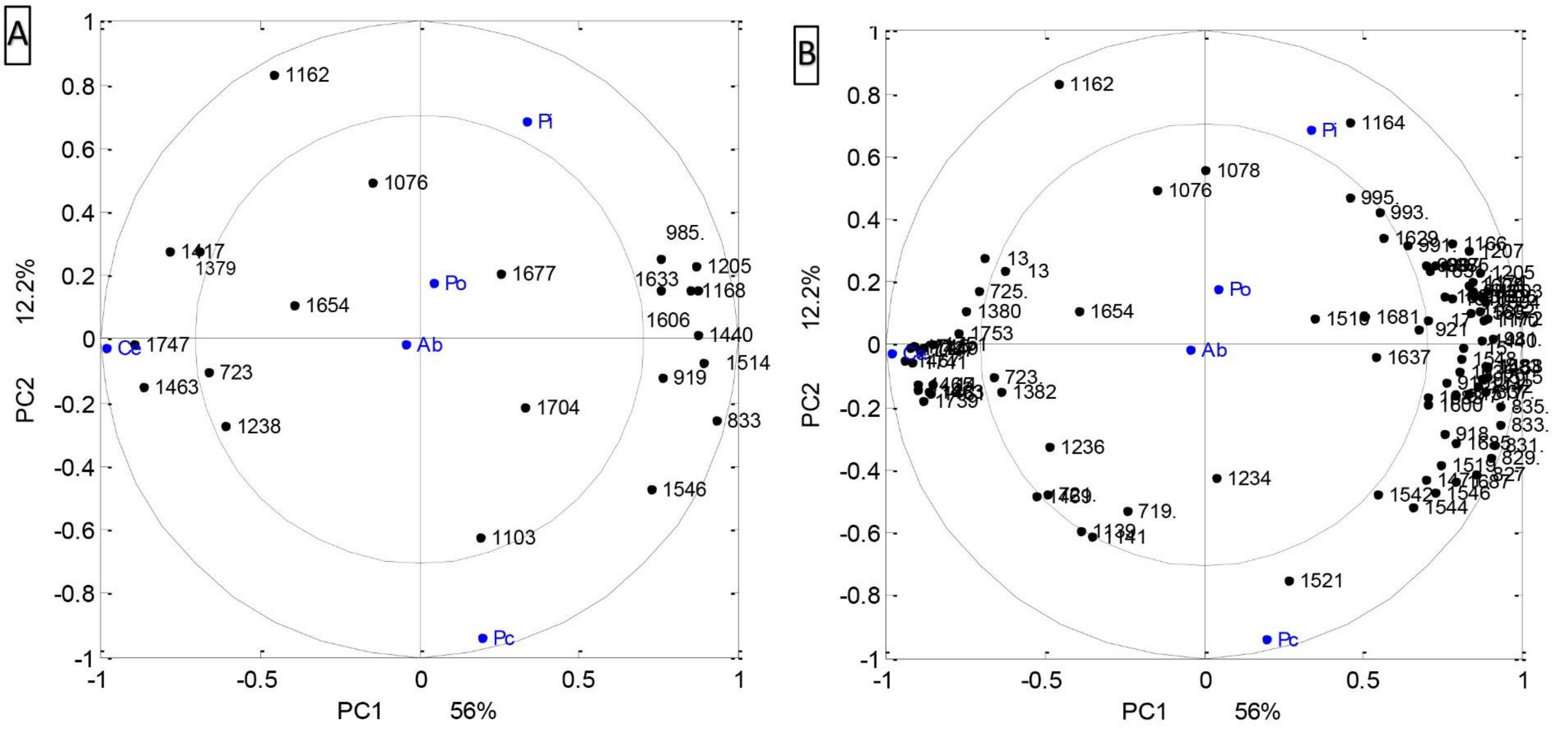

Fig. C (Part 1) CPCA correlation loading plots for the first two principal components. Variables related to transmission FTIR spectroscopy of KBr pellets (**KBR**) are presented in black color; **(A)** plot for the selected variables, and **(B)** plot for all variables. Design variables related to plant genera are presented in blue color: *Abies* (Ab), *Cedrus* (Ce), *Picea* (Pc), *Pinus* (Pi), *Podocarpus* (Po).

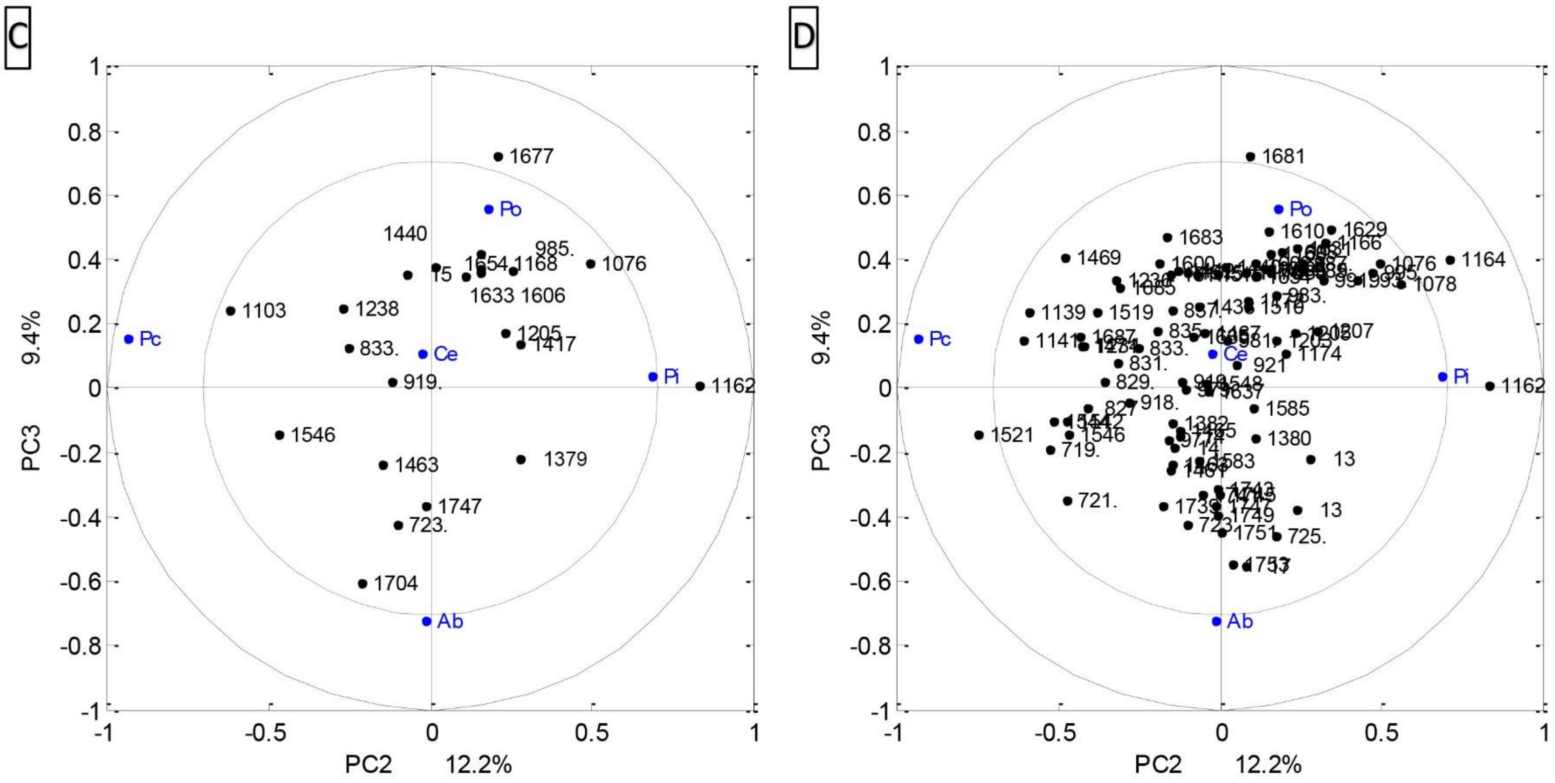

Fig. C (Part 2) CPCA correlation loading plots for the second and the third principal components. Variables related to transmission FTIR spectroscopy of KBr pellets (**KBR**) are presented in black color; **(C)** plot for the selected variables, and **(D)** plot for all variables. Design variables related to plant genera are presented in blue color: *Abies* (Ab), *Cedrus* (Ce), *Picea* (Pc), *Pinus* (Pi), *Podocarpus* (Po).

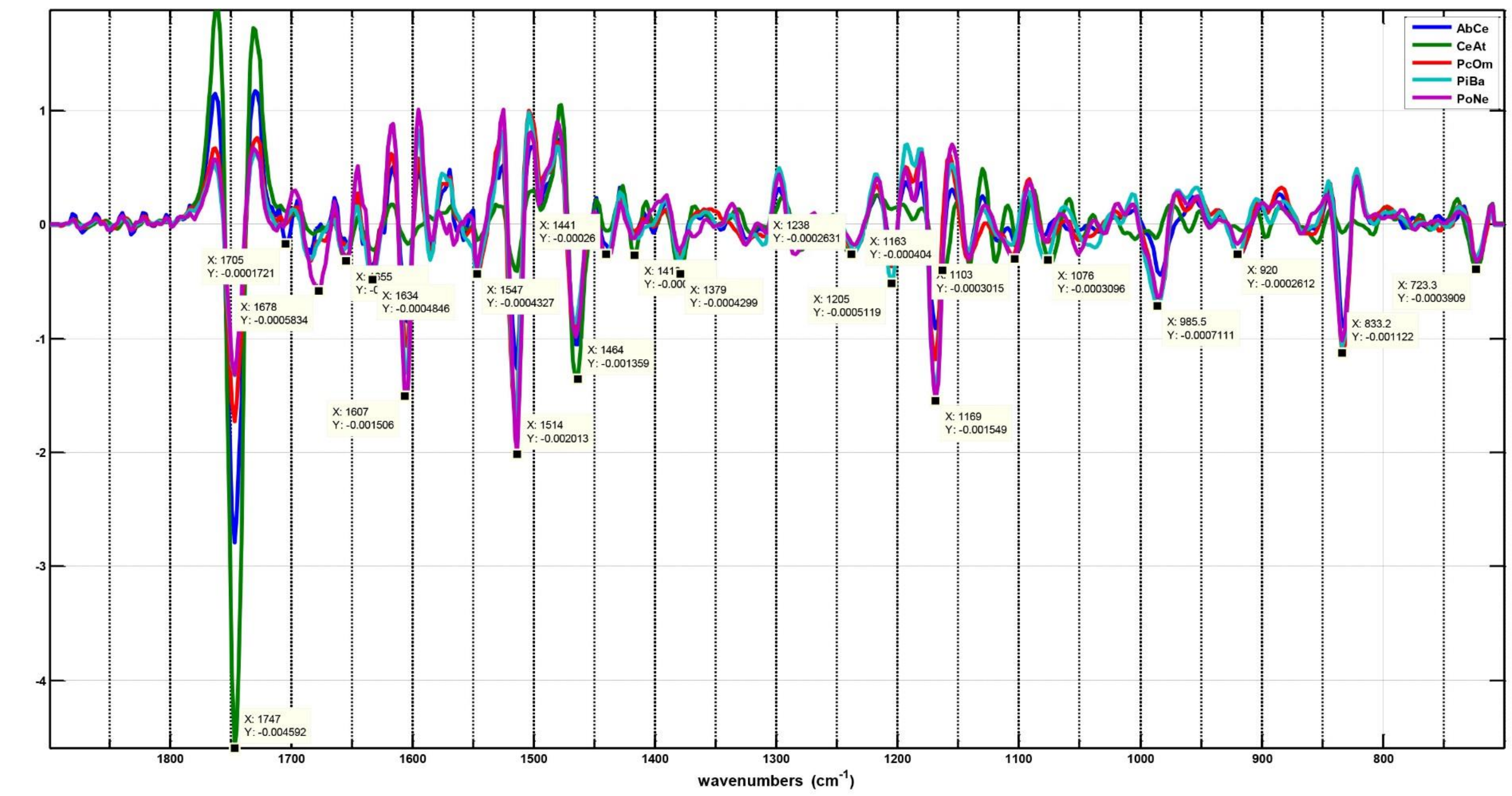

Fig. C (Part 3) Second-derivative and EMSC corrected spectra obtained by transmission FTIR spectroscopy of KBr pellets (**KBR**). Average spectra of five representative species are presented: *Abies cephalonica* (AbCe), *Cedrus atlantica* (CeAt), *Picea omorika* (PcOm), *Pinus banksiana* (PiBa), *Podocarpus neriifolius* (PoNe). The selected vibrational bands associated to the CPCA correlation loading plots on Figures S3 are marked.

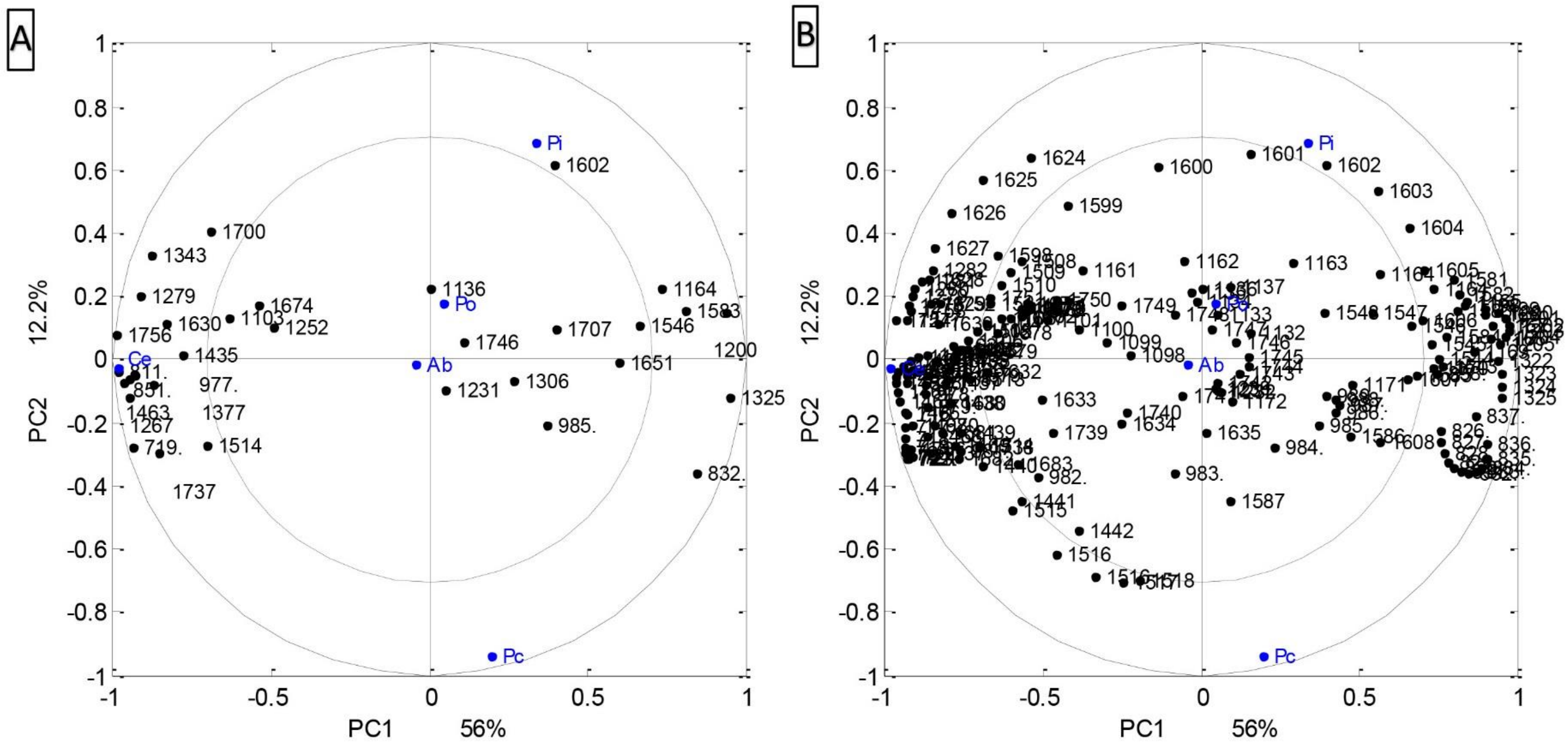

Fig. D (Part 1) CPCA correlation loading plots for the first two principal components. Variables related to transmission FTIR microspectroscopy of pollen multigrain (**MGR**) are presented in black color; **(A)** plot for the selected variables, and **(B)** plot for all variables. Design variables related to plant genera are presented in blue color: *Abies* (Ab), *Cedrus* (Ce), *Picea* (Pc), *Pinus* (Pi), *Podocarpus* (Po).

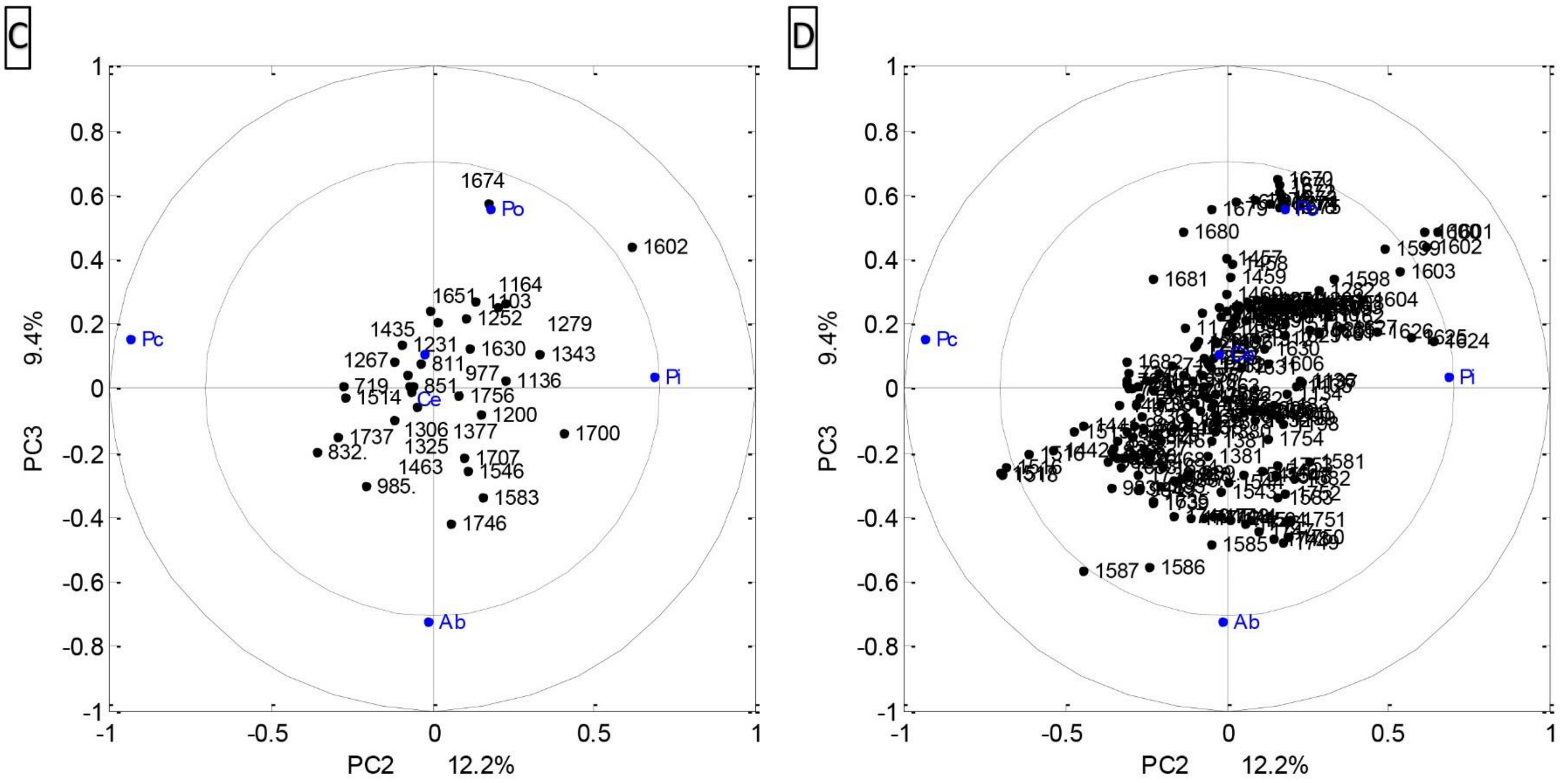

Fig. D (Part 2) CPCA correlation loading plots for the second and the third principal components. Variables related to transmission FTIR microspectroscopy of pollen multigrain (**MGR**) are presented in black color; **(C)** plot for the selected variables, and **(D)** plot for all variables. Design variables related to plant genera are presented in blue color: *Abies* (Ab), *Cedrus* (Ce), *Picea* (Pc), *Pinus* (Pi), *Podocarpus* (Po).

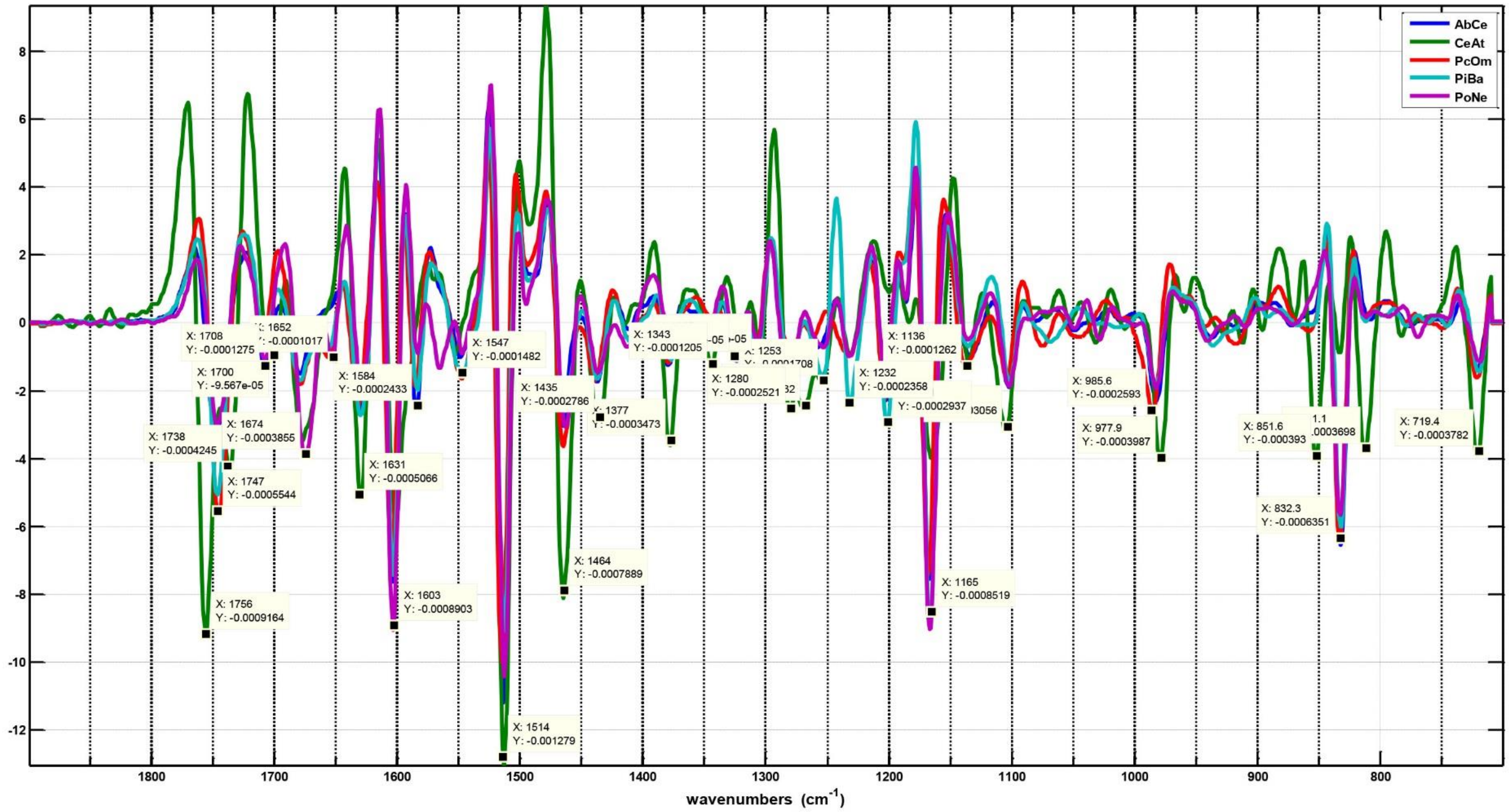

Fig. D (Part 3) Second-derivative and EMSC corrected spectra obtained by transmission FTIR microspectroscopy of pollen multigrain (**MGR**). Average spectra of five representative species are presented: *Abies cephalonica* (AbCe), *Cedrus atlantica* (CeAt), *Picea omorika* (PcOm), *Pinus banksiana* (PiBa), *Podocarpus neriifolius* (PoNe). The selected vibrational bands associated to the CPCA correlation loading plots on Figures S4 are marked.

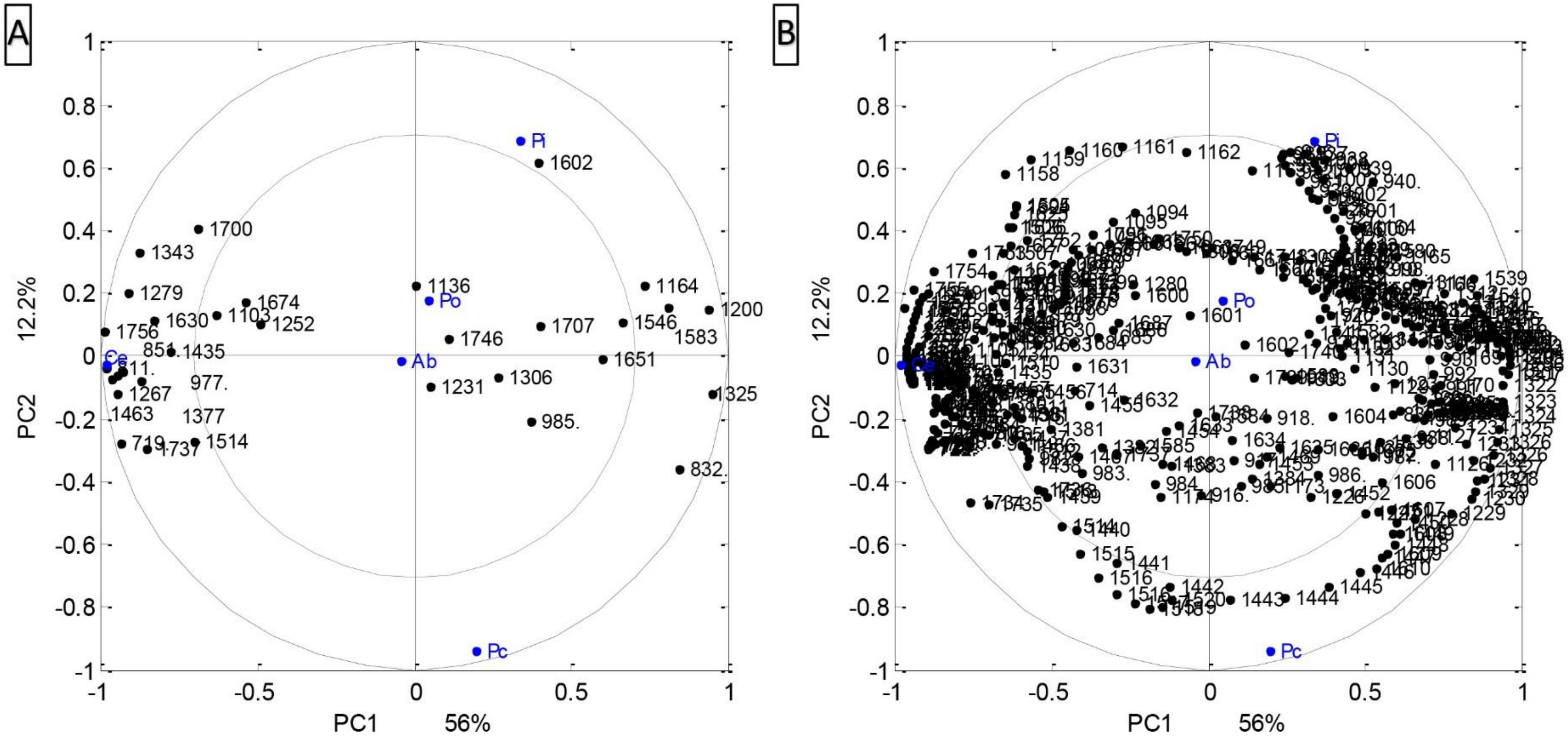

Fig. E (Part 1) CPCA correlation loading plots for the first two principal components. Variables related to transmission FTIR microspectroscopy of single pollen grain (**SGR**) are presented in black color; **(A)** plot for the selected variables, and **(B)** plot for all variables. Design variables related to plant genera are presented in blue color: *Abies* (Ab), *Cedrus* (Ce), *Picea* (Pc), *Pinus* (Pi), *Podocarpus* (Po).

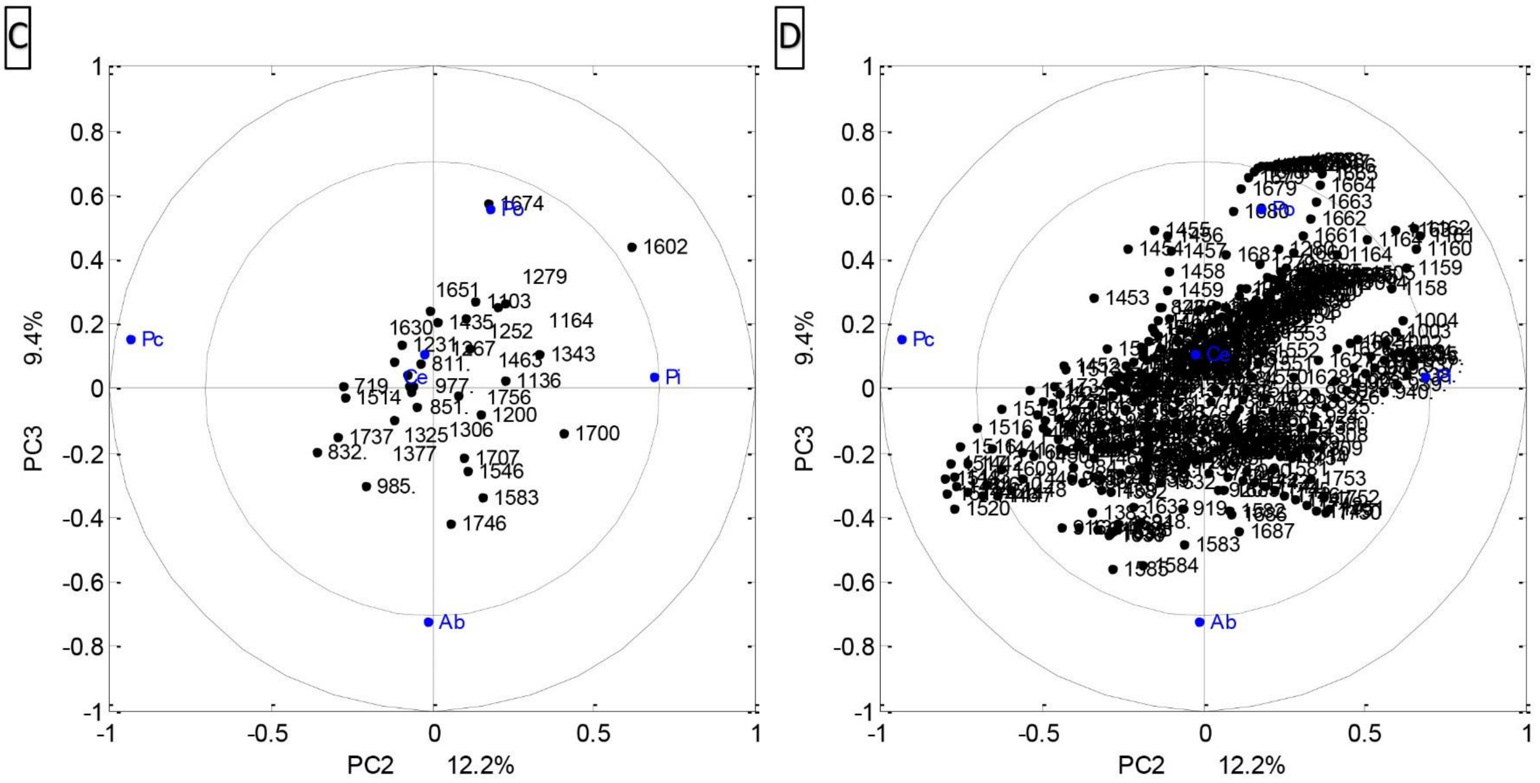

Fig. E (Part 2) CPCA correlation loading plots for the second and the third principal components. Variables related to transmission FTIR microspectroscopy of single pollen grain (**SGR**) are presented in black color; **(C)** plot for the selected variables, and **(D)** plot for all variables. Design variables related to plant genera are presented in blue color: *Abies* (Ab), *Cedrus* (Ce), *Picea* (Pc), *Pinus* (Pi), *Podocarpus* (Po).

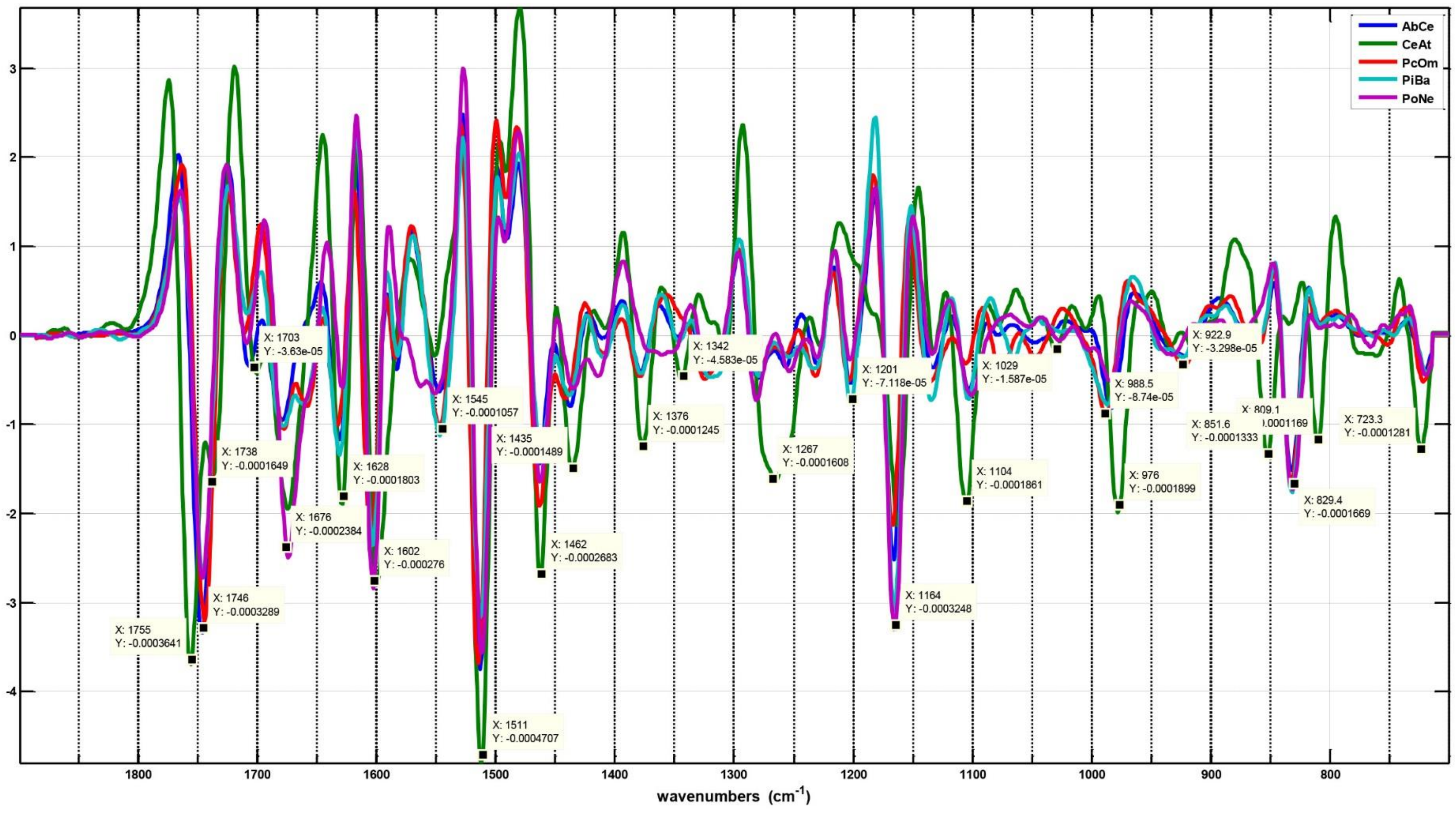

Fig. E (Part 3) Second-derivative and EMSC corrected spectra obtained by transmission FTIR microspectroscopy of single pollen grain (**SGR**). Average spectra of five representative species are presented: *Abies cephalonica* (AbCe), *Cedrus atlantica* (CeAt), *Picea omorika* (PcOm), *Pinus banksiana* (PiBa), *Podocarpus neriifolius* (PoNe). The selected vibrational bands associated to the CPCA correlation loading plots on Figures S5 are marked.

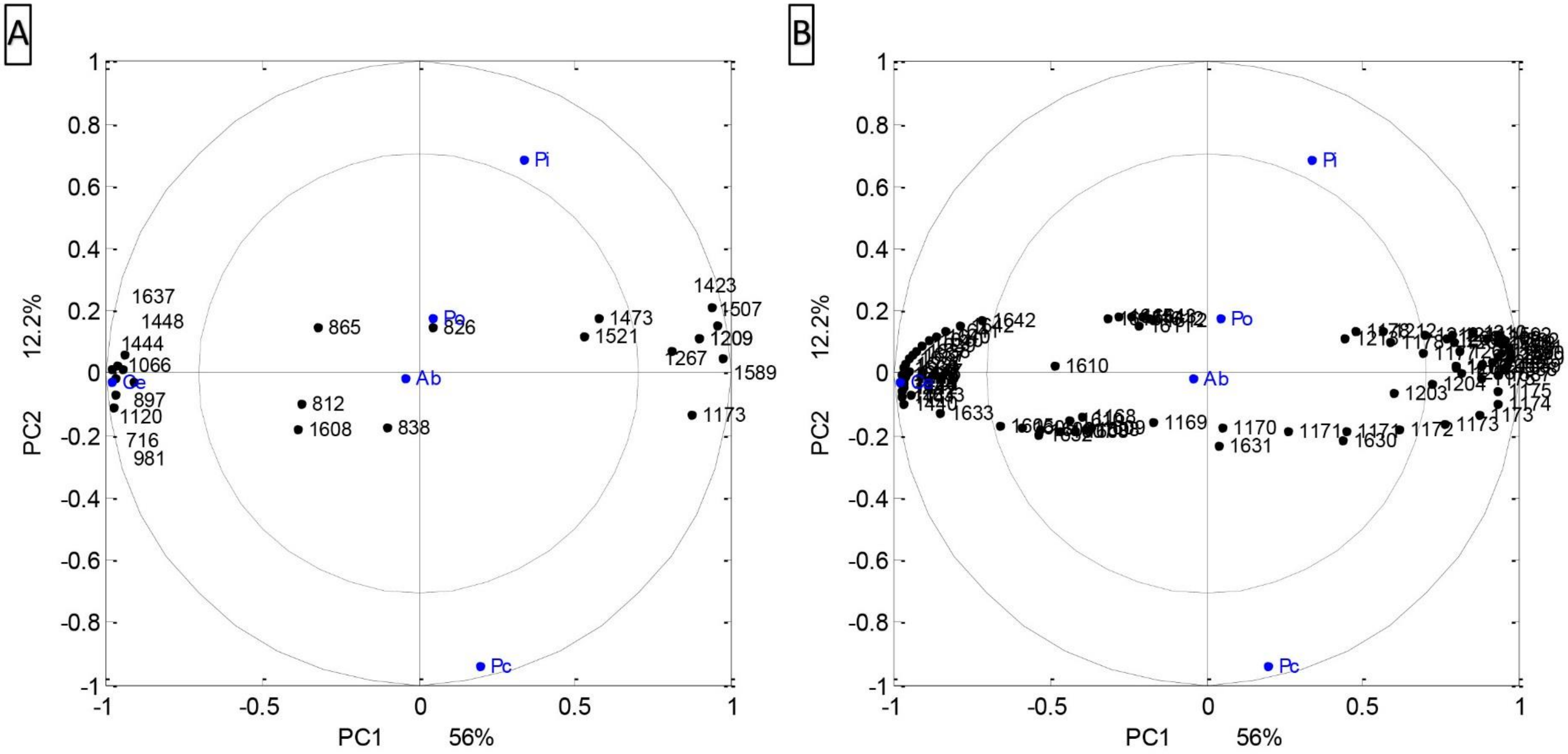

Fig. F (Part 1) CPCA correlation loading plots for the first two principal components. Variables related to Raman microspectroscopy measurements of saccus part of pollen grain (**RMS**) are presented in black color; **(A)** plot for the selected variables, and **(B)** plot for all variables. Design variables related to plant genera are presented in blue color: *Abies* (Ab), *Cedrus* (Ce), *Picea* (Pc), *Pinus* (Pi), *Podocarpus* (Po).

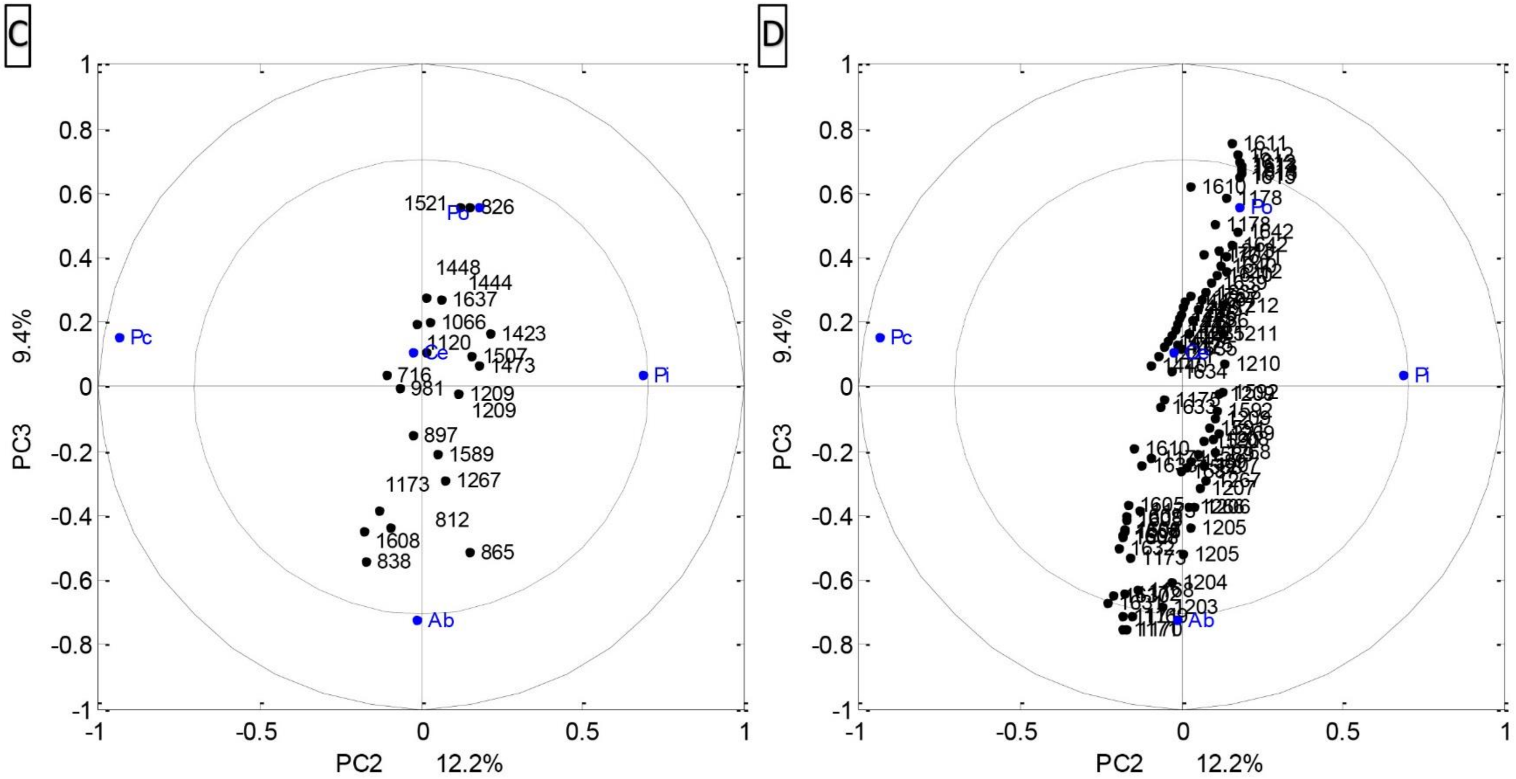

Fig. F (Part 2) CPCA correlation loading plots for the second and the third principal components. Variables related to Raman microspectroscopy measurements of saccus part of pollen grain (**RMS**) are presented in black color; **(C)** plot for the selected variables, and **(D)** plot for all variables. Design variables related to plant genera are presented in blue color: *Abies* (Ab), *Cedrus* (Ce), *Picea* (Pc), *Pinus* (Pi), *Podocarpus* (Po).

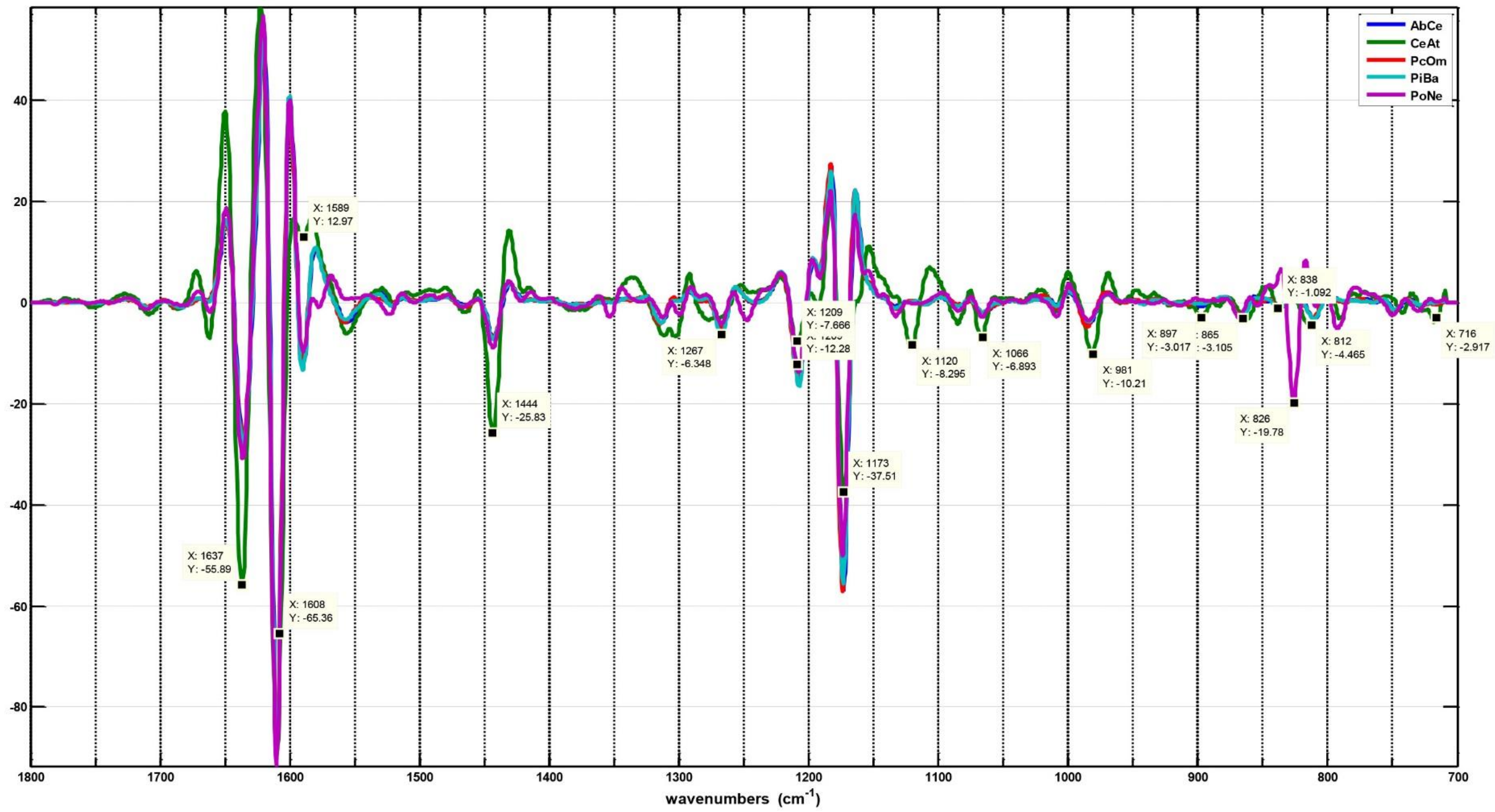

Fig. F (Part 3) Second-derivative and EMSC corrected spectra obtained by Raman microspectroscopy measurements of saccus part of pollen grain (**RMS**). Average spectra of five representative species are presented: *Abies cephalonica* (AbCe), *Cedrus atlantica* (CeAt), *Picea omorika* (PcOm), *Pinus banksiana* (PiBa), *Podocarpus neriifolius* (PoNe). The selected vibrational bands associated to the CPCA correlation loading plots on Figures S6 are marked.

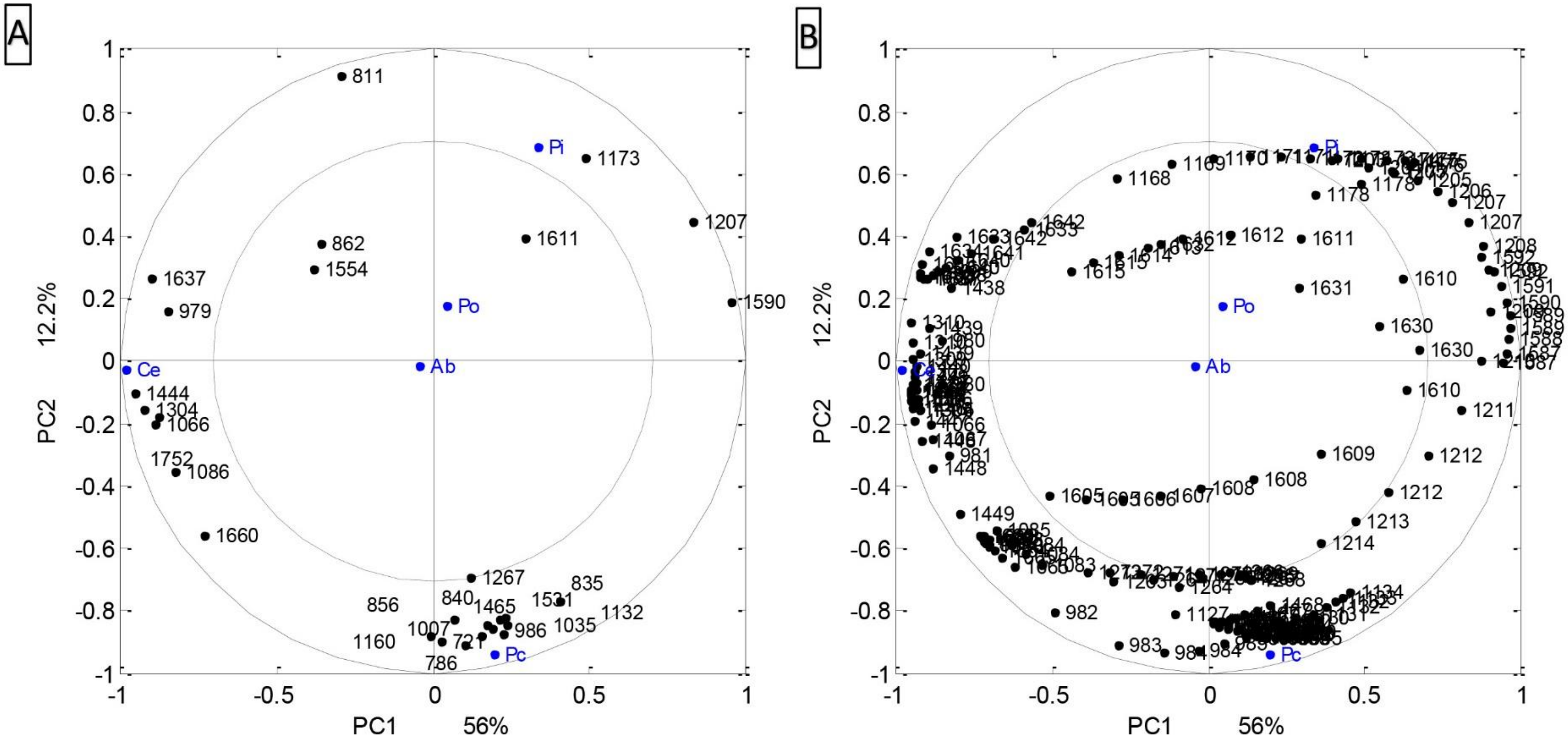

Fig. G (Part 1) CPCA correlation loading plots for the first two principal components. Variables related to Raman microspectroscopy measurements of corpus part of pollen grain (**RMC**) are presented in black color; **(A)** plot for the selected variables, and **(B)** plot for all variables. Design variables related to plant genera are presented in blue color: *Abies* (Ab), *Cedrus* (Ce), *Picea* (Pc), *Pinus* (Pi), *Podocarpus* (Po).

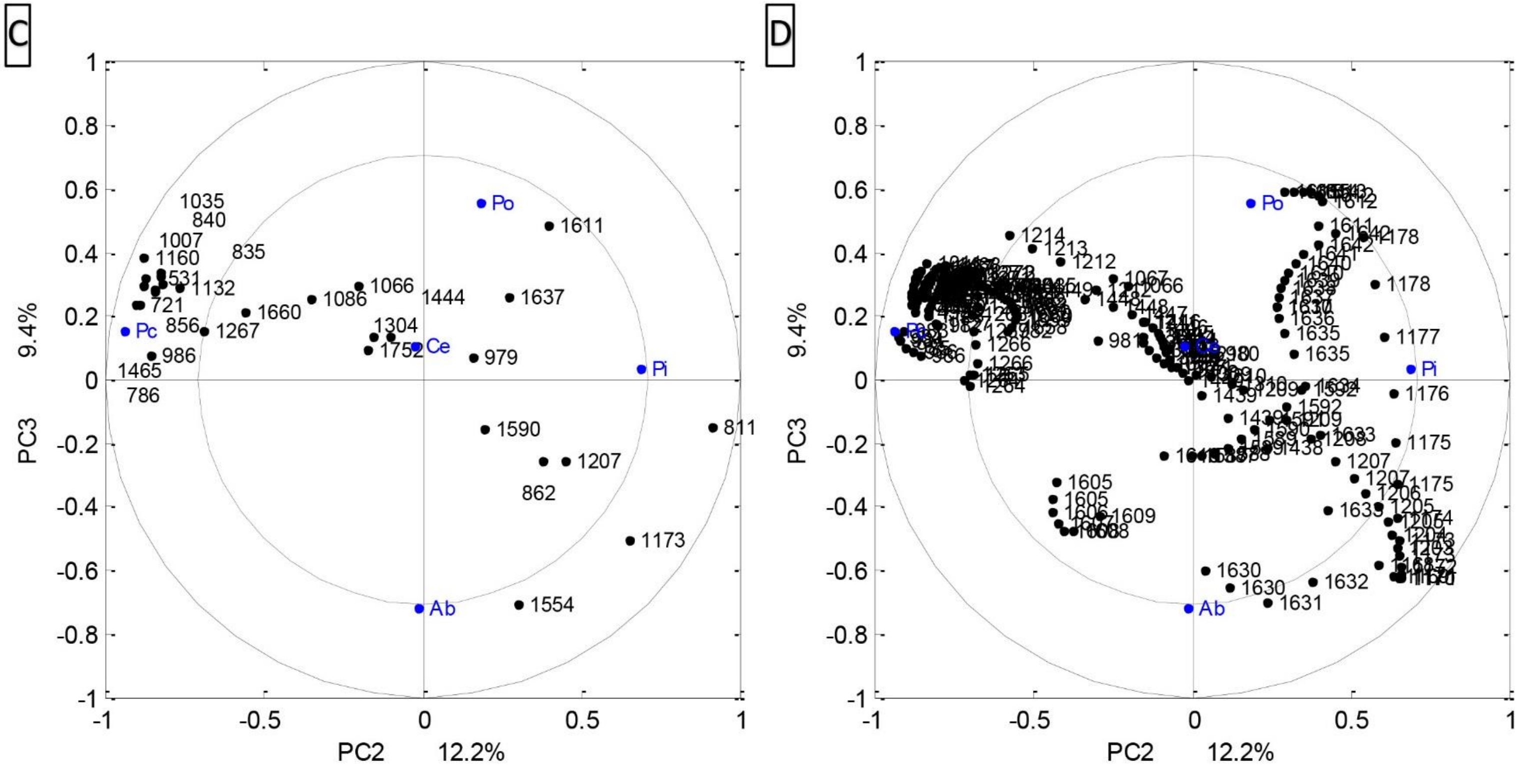

Fig. G (Part 2) CPCA correlation loading plots for the second and the third principal components. Variables related to Raman microspectroscopy measurements of corpus part of pollen grain (**RMC**) are presented in black color; **(C)** plot for the selected variables, and **(D)** plot for all variables. Design variables related to plant genera are presented in blue color: *Abies* (Ab), *Cedrus* (Ce), *Picea* (Pc), *Pinus* (Pi), *Podocarpus* (Po).

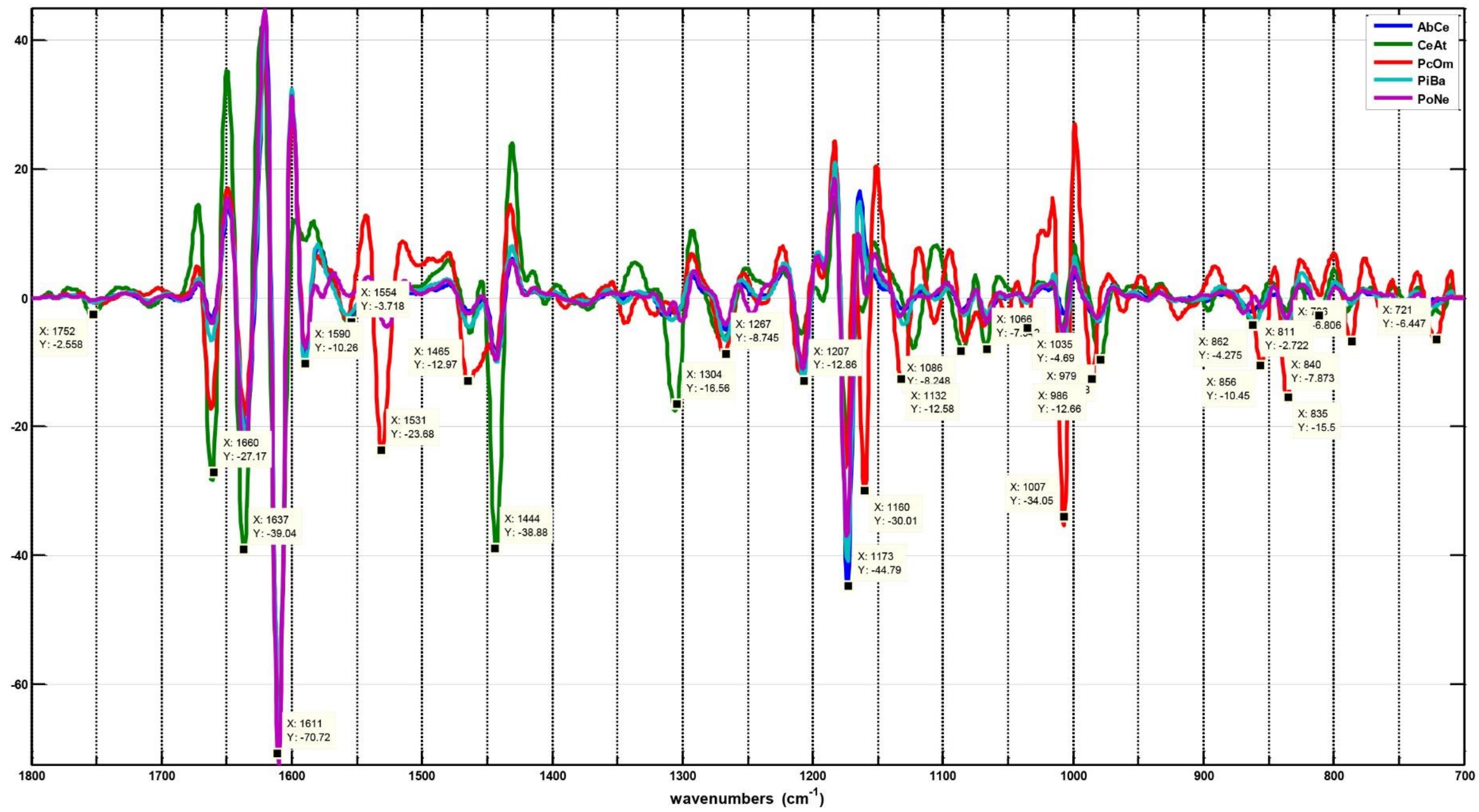

Fig. G (Part 3) Second-derivative and EMSC corrected spectra obtained by Raman microspectroscopy measurements of corpus part of pollen grain (**RMC**). Average spectra of five representative species are presented: *Abies cephalonica* (AbCe), *Cedrus atlantica* (CeAt), *Picea omorika* (PcOm), *Pinus banksiana* (PiBa), *Podocarpus neriifolius* (PoNe). The selected vibrational bands associated to the CPCA correlation loading plots on Figures S7 are marked.

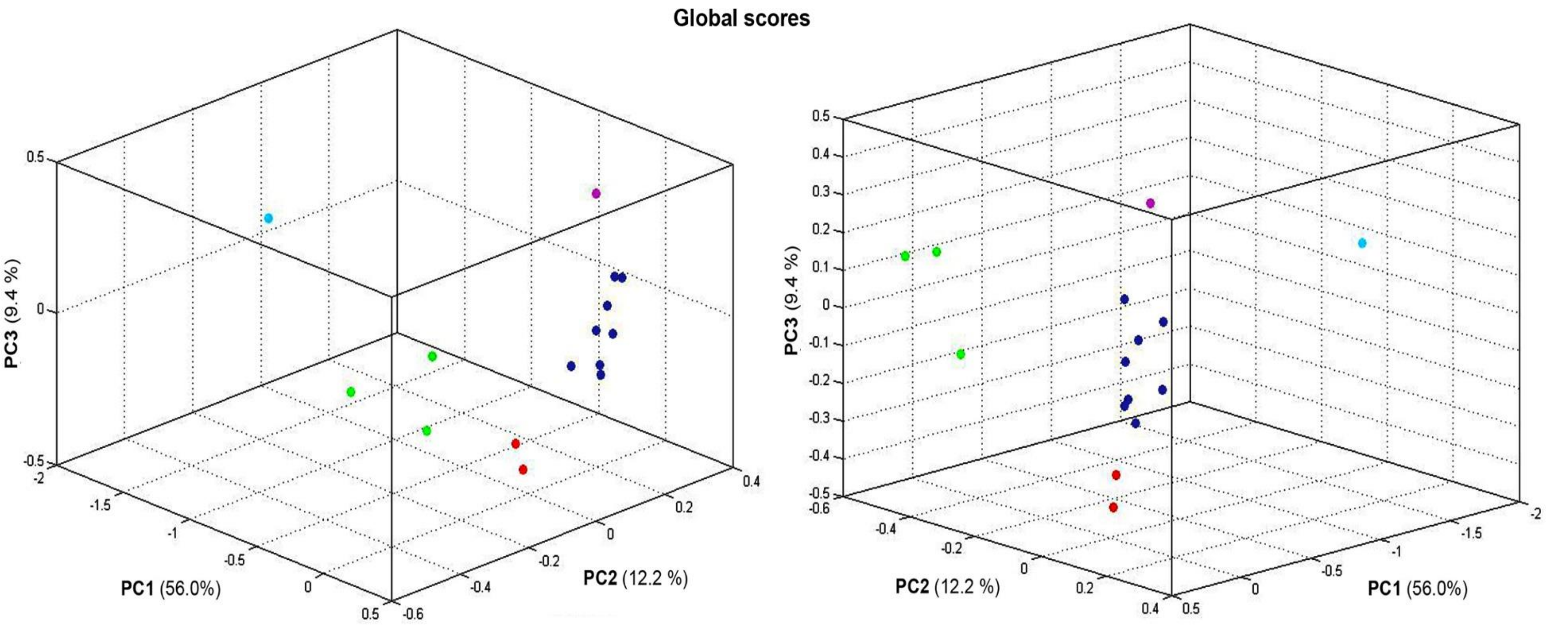

Fig. H 3D presentation of global scores of consensus principal component analysis (CPCA). Markings are labelled in accordance to pollen genus: *Abies* (red), *Cedrus* (blue), *Picea* (green), *Pinus* (dark yellow), and *Podocarpus* (violet). The percent variances for the first five PCs are 56.0, 12.2, 9.4, 7.5 and 5.1.

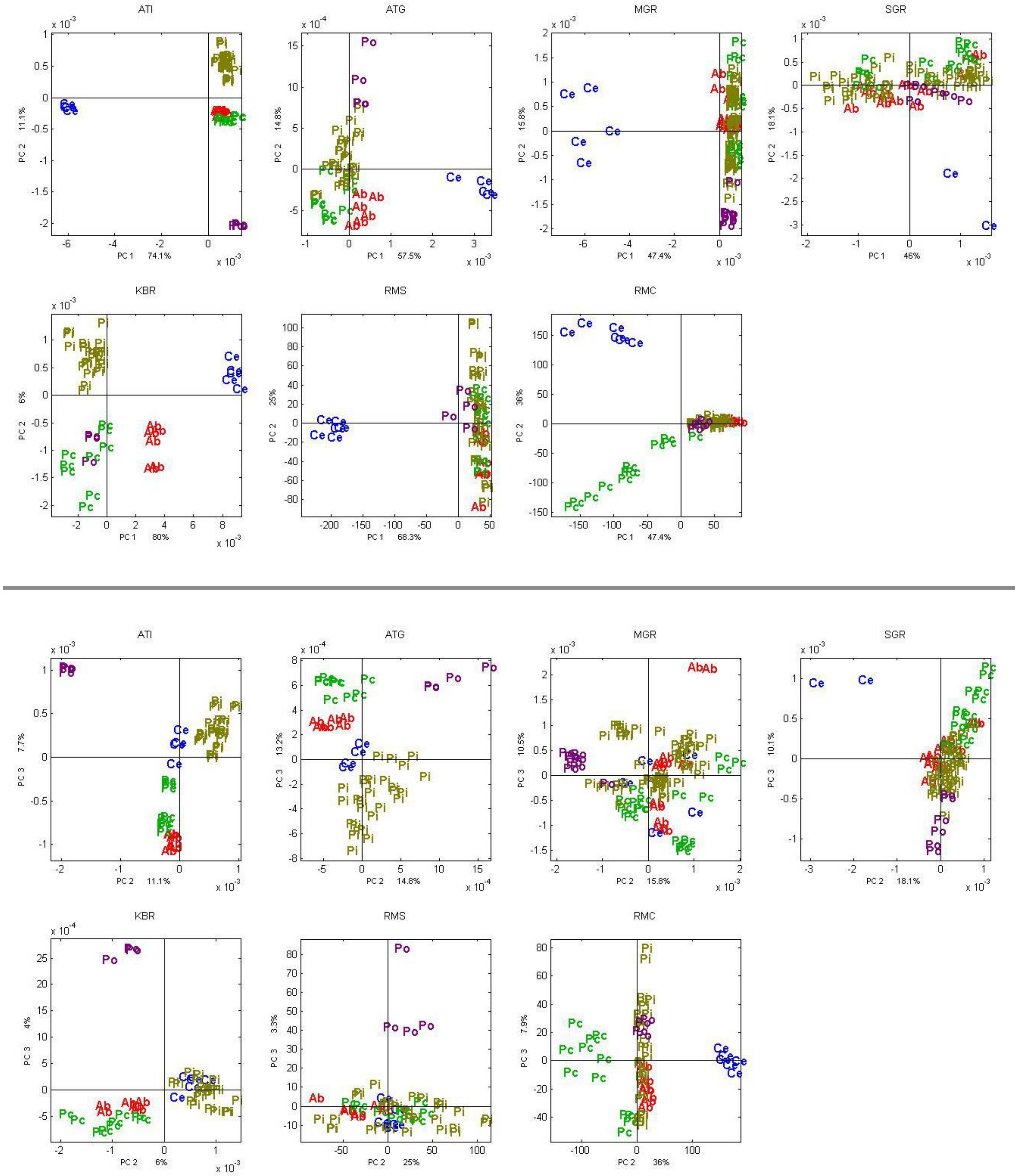

Fig. I PCA score plots performed on individual spectral data blocks: 1) ATI (ATR-FTIR of intact pollen), 2) ATG (ATR-FTIR of ground pollen), 3) MGR (transmission FTIR microspectroscopy of multigrain), 4) SGR (transmission FTIR microspectroscopy of single grain), 5) KBR (transmission FTIR of KBr pellets), 6) RMC (Raman of corpus region) and 7) RMS (Raman of saccus region). Samples are labelled in accordance to pollen genus: *Abies* (Ab, red), *Cedrus* (Ce, blue), *Picea* (Pc, green), *Pinus* (Pi, dark yellow), *Podocarpus* (Po, violet).

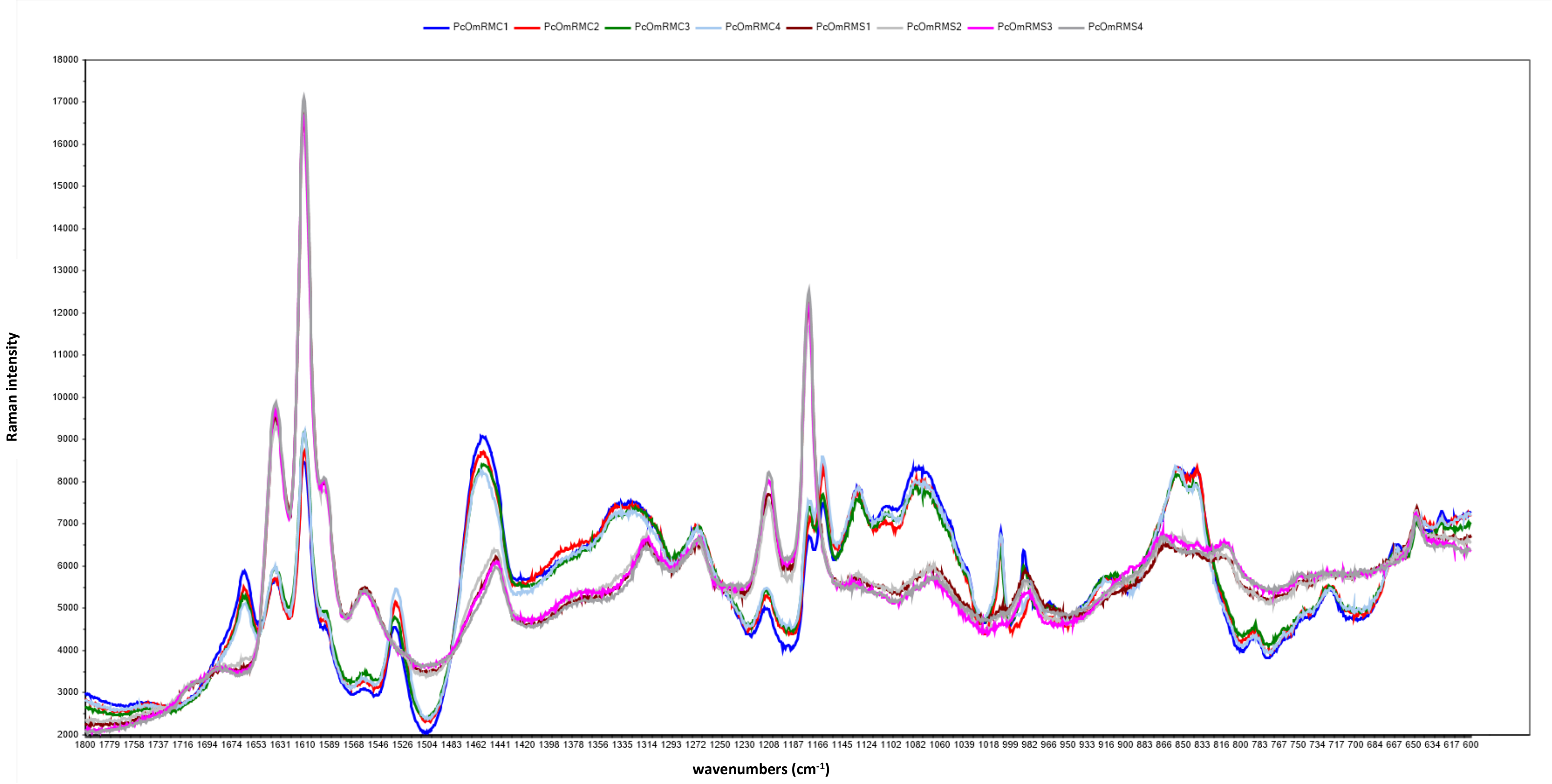

Fig. J Raman spectra of representative samples of *Picea omorika* pollen. The spectral set consists of EMSC normalized spectra of measurements of corpus region (RMC), and saccus region (RMS), with four spectra per region.

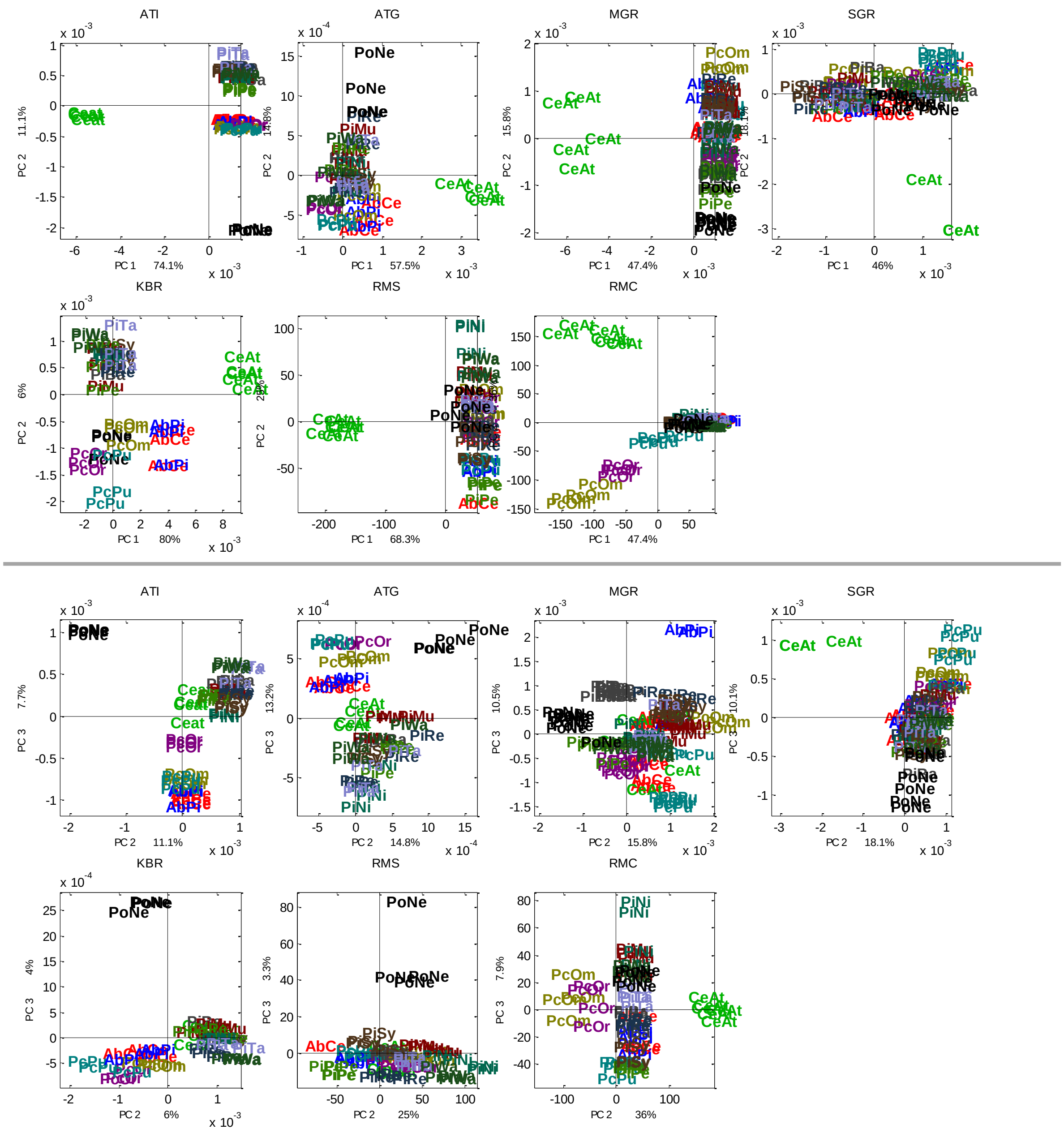

Fig. K PCA score plots performed on individual spectral data blocks: 1) ATI (ATR-FTIR of intact pollen), 2) ATG (ATR-FTIR of ground pollen), 3) MGR (transmission FTIR microspectroscopy of multigrain), 4) SGR (transmission FTIR microspectroscopy of single grain), 5) KBR (transmission FTIR of KBr pellets), 6) RMC (Raman of corpus region) and 7) RMS (Raman of saccus region). Samples are labelled in accordance to pollen species: *Abies cephalonica* (AbCe), *Cedrus atlantica* (CeAt), *Picea omorika* (PcOm), *Picea orientalis* (PcOr), *Picea pungens* (PcPu), *Pinus banksiana* (PiBa), *Pinus mugo* (PiMu), *Pinus nigra* (PiNi), *Pinus peuce* (PiPe), *Pinus resinosa* (PiRe), *Pinus sylvestris* (PiSy), *Pinus tabuliformis* (PiTa), *Pinus wallichiana* (PiWa),*Podocarpus neriifilious* (PoNe).

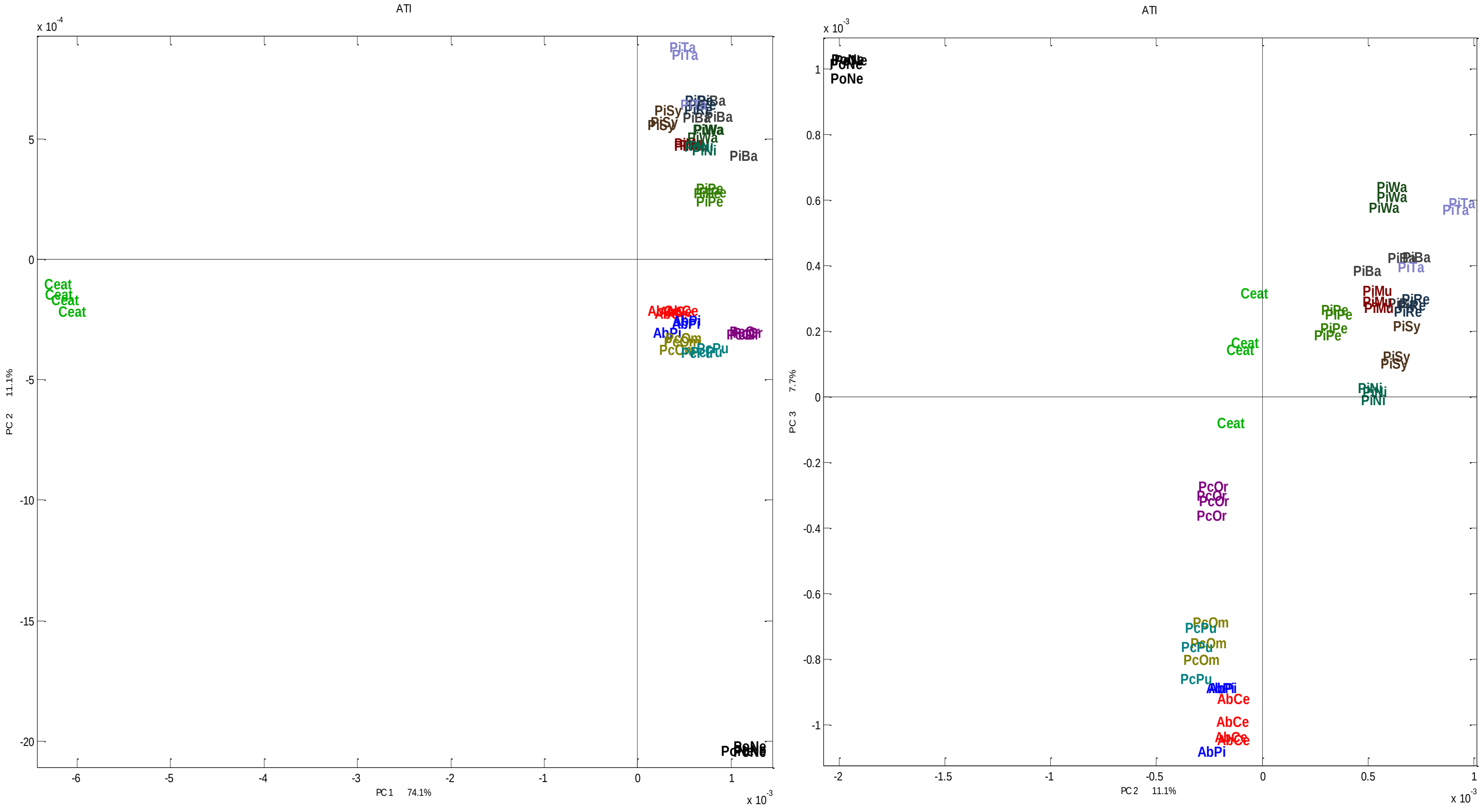

Fig. L PCA score plots performed on individual spectral data blocks: ATI (ATR-FTIR of intact pollen). Samples are labelled in accordance to pollen species: *Abies cephalonica* (AbCe), *Cedrus atlantica* (CeAt), *Picea omorika* (PcOm), *Picea orientalis* (PcOr), *Picea pungens* (PcPu), *Pinus banksiana* (PiBa), *Pinus mugo* (PiMu), *Pinus nigra* (PiNi), *Pinus peuce* (PiPe), *Pinus resinosa* (PiRe), *Pinus sylvestris* (PiSy), *Pinus tabuliformis* (PiTa), *Pinus wallichiana* (PiWa),*Podocarpus neriifilious* (PoNe).

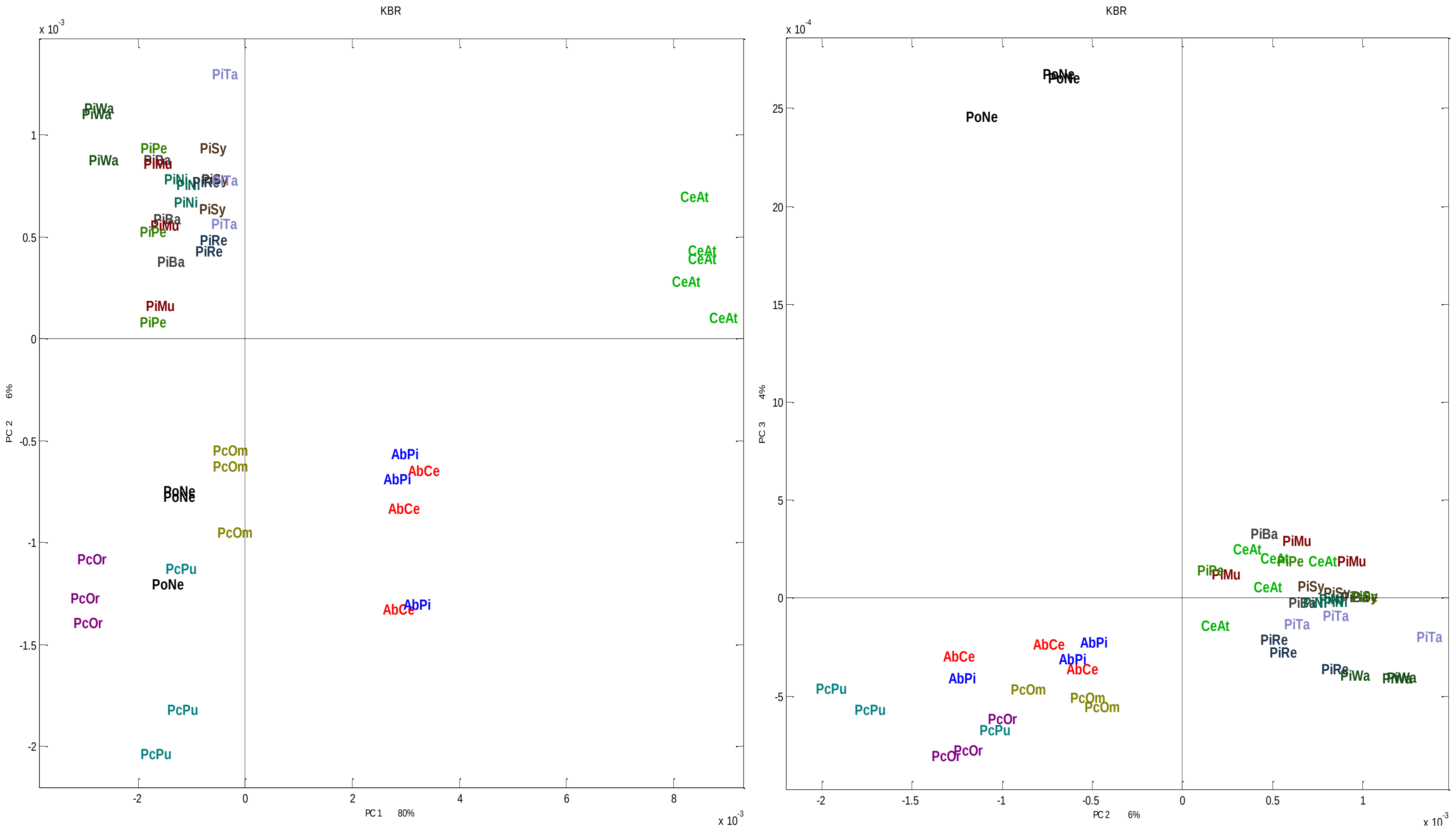

Fig. M PCA score plots performed on individual spectral data blocks: KBR (transmission FTIR of KBr pellets). Samples are labelled in accordance to pollen species: *Abies cephalonica* (AbCe), *Cedrus atlantica* (CeAt), *Picea omorika* (PcOm), *Picea orientalis* (PcOr), *Picea pungens* (PcPu), *Pinus banksiana* (PiBa), *Pinus mugo* (PiMu), *Pinus nigra* (PiNi), *Pinus peuce* (PiPe), *Pinus resinosa* (PiRe), *Pinus sylvestris* (PiSy), *Pinus tabuliformis* (PiTa), *Pinus wallichiana* (PiWa),*Podocarpus neriifilious* (PoNe).

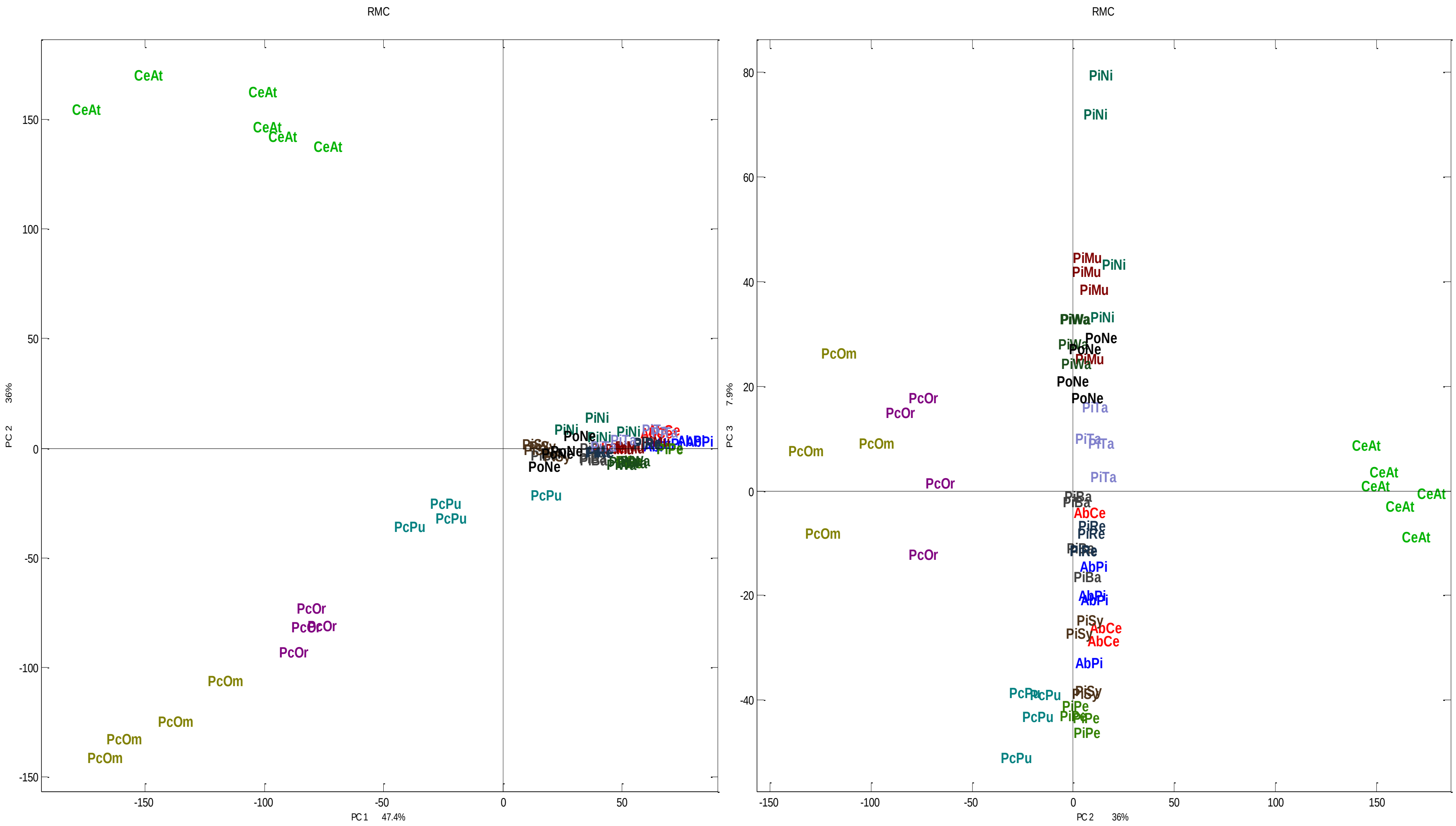

Fig. N PCA score plots performed on individual spectral data blocks: RMC (Raman spectra of corpus region). Samples are labelled in accordance to pollen species: *Abies cephalonica* (AbCe), *Cedrus atlantica* (CeAt), *Picea omorika* (PcOm), *Picea orientalis* (PcOr), *Picea pungens* (PcPu), *Pinus banksiana* (PiBa), *Pinus mugo* (PiMu), *Pinus nigra* (PiNi), *Pinus peuce* (PiPe), *Pinus resinosa* (PiRe), *Pinus sylvestris* (PiSy), *Pinus tabuliformis* (PiTa), *Pinus wallichiana* (PiWa),*Podocarpus neriifilious* (PoNe).

Table A Variability measurements within replicates[§] of *Pinus* genus.

**IR Region**	**Species**								**Genus**
**1200-700 cm^{-1}** **(1-*PCC**)x10^{-4}**	***Pinus banksiana***	***Pinus peuce***	***Pinus mugo***	***Pinus nigra***	***Pinus resinosa***	***Pinus sylvetris***	***Pinus tabuliformis***	***Pinus, wallichiana***	***Pinus***
**ATI**	0.003681	0.000889	0.001097	0.001414	0.001061	0.001272	0.001957	0.000922	0.0043
**ATG**	0.008495	0.005373	0.024527	0.030032	0.019066	0.010331	0.038434	0.015859	0.0381
**KBR**	0.074957	0.096663	0.029511	0.168076	0.177112	0.07285	0.088509	0.064469	0.1760
**MGR**	0.006705	0.002553	0.009297	0.001983	0.012672	0.006115	0.001965	0.004316	0.0180
**SGR**	0.269506	0.074953	0.235117	0.080506	0.11851	0.254767	0.085362	0.068036	0.1927
**RMS**	0.00051	0.000404	0.001007	0.002186	0.000739	0.001633	0.000143	0.000233	0.0070
**RMC**	0.002131	0.001705	0.001667	0.004697	0.002735	0.001638	0.00268	0.000619	0.0108

**IR Region**	**Species**								**Genus**
**1800-1500 cm^{-1}** **(1-*PCC**)x10^{-4}**	***Pinus banksiana***	***Pinus peuce***	***Pinus mugo***	***Pinus nigra***	***Pinus resinosa***	***Pinus sylvetris***	***Pinus tabuliformis***	***Pinus wallichiana***	***Pinus***
**ATI**	0.005322	0.000278	0.000382	0.009551	0.000311	0.003848	0.009442	0.000768	0.0067
**ATG**	0.026859	0.099302	0.095689	0.122511	0.031509	0.002885	0.026689	0.00311	0.0698
**KBR**	0.040537	0.073159	0.046467	0.00788	0.021484	0.019477	6.60E-02	0.01467	0.0487
**MGR**	0.008991	0.038083	0.004665	0.020438	0.023404	0.006171	0.012604	0.009468	0.0293
**SGR**	0.134267	0.043698	0.068565	0.109443	0.023178	0.107776	0.076228	0.016804	0.1057
**RMS**	0.000186	0.000219	0.000585	0.001669	0.000926	0.001032	8.40E-05	0.000381	0.0081
**RMC**	0.000538	0.000187	0.000365	0.002432	0.000268	0.000659	0.000346	0.000137	0.0074

*Pearson Correlation Coefficient (PCC)

[§]Replicate spectra were obtained from same sample measured as at least three times.

Table B Variability measurements within replicates§ of *Picea* genus

**IR Region**	**Species**			**Genus**
**1200-700 cm^{-1} (1-*PCC**)x10^{-4}**	***Picea omorika***	***Picea oritentalis***	***Picea pungens***	***Picea***
**ATI**	0.00109	0.00165	0.00308	0.00360
**ATG**	0.00858	0.01830	0.01088	0.00287
**KBR**	0.26673	0.06914	0.25413	0.37640
**MGR**	0.00377	0.00272	0.00659	0.01660
**SGR**	0.08598	0.05455	0.01603	0.06600
**RMS**	0.00164	0.00078	0.00030	0.00380
**RMC**	0.02821	0.01491	0.01526	0.06600

**IR Region**	**Species**			**Genus**
**1800-1500 cm^{-1} (1-*PCC**)x10^{-4}**	***Picea omorika***	***Picea oritentalis***	***Picea pungens***	***Picea***
**ATI**	0.00065	0.00097	0.00223	0.00460
**ATG**	0.00537	0.22381	0.02451	0.10210
**KBR**	0.02929	0.01872	0.11992	0.09470
**MGR**	0.00152	0.27308	0.01386	0.11870
**SGR**	0.05143	0.04629	0.00413	0.05790
**RMS**	0.00069	0.00103	0.00016	0.00350
**RMC**	0.00303	0.00325	0.00145	0.00960

*Pearson Correlation Coefficient (PCC)

§Replicate spectra were obtained from same sample measured as at least three times.

Reproducibility and variability of vibrational spectroscopic measurements was estimated by Pearson's correlation coefficient (*PCC*). For the sake of clarity, due to attained *PCC*-values, which were very close to one, results were displayed as 1-PCC.The closer the obtained values are to zero, the lower the variability in the respective data set. The variability test was performed for the two different spectral regions. The spectra were transformed to second derivative form by Savitzky-Golay (SG) algorithm algorithm followed by Extended multiplicative signal correction (EMSC) pre-processing, same as performed in Consensus principal component analysis (CPCA).
